# Renal microcirculation and mechanisms in diabetic kidney disease

**DOI:** 10.3389/fendo.2025.1580608

**Published:** 2025-06-05

**Authors:** Xing Hang, Jiang Ma, Yu Wei, Yayun Wang, Xiaoyu Zang, Pengfei Xie, Lili Zhang, Linhua Zhao

**Affiliations:** ^1^ Institute of Metabolic Diseases, Guang’anmen Hospital, China Academy of Chinese Medical Sciences, Beijing, China; ^2^ Beijing University of Chinese Medicine, Beijing, China; ^3^ Guangwai Hospital, Beijing, China; ^4^ Changchun University of Chinese Medicine, Changchun, China

**Keywords:** diabetic kidney disease, renal microcirculation, mechanisms, animal model, assessment techniques

## Abstract

Diabetic kidney disease (DKD), a severe and long-term complication of diabetes, is a microcirculatory pathology influenced by diabetes-related factors that affects hundreds of millions of people worldwide. DKD is characterized by proteinuria, glomerular injury, and renal fibrosis, ultimately leading to end-stage renal disease. Its pathogenesis is complex and involves multiple cellular and molecular mechanisms. Microcirculatory disorders form the fundamental pathological basis of DKD. These disorders are primarily manifested through changes in the number and structure of renal microvessels, alterations in renal hemodynamics, formation of renal thrombi, glomerular endothelial cell dysfunction, and associated lesions in podocytes and mesangial cells. This article focuses on renal microangiopathy and glomerular endothelial cell (GEC) dysfunction, summarizing the mechanisms associated with microcirculatory lesions in DKD, including nitric oxide (NO), advanced glycation end-products (AGEs), vascular endothelial growth factor (VEGF), the renin-angiotensin-aldosterone system (RAAS), reactive oxygen species (ROS), the NLRP3 inflammasome, protein kinase C (PKC), epidermal growth factor receptor (EGFR), and platelet-derived growth factor (PDGF). Additionally, we briefly introduce the characteristics of DKD animal models in terms of renal microcirculation and discuss the application of relevant technological tools in studying microcirculatory lesions in DKD.

## 1 Introduction

In 2021, an estimated 536 million individuals worldwide had diabetes, with 50% of hemodialysis patients affected. The global diabetic population is projected to reach 783 million by 2045, posing a significant threat to public health (1). Diabetic kidney disease (DKD) is a chronic condition that arises as a result of diabetes mellitus. It is one of the most common microvascular complications of diabetes mellitus and represents a major cause of end-stage renal disease (ESRD), accounting for about 30%-50% of ESRD cases worldwide (2). In addition, a report indicates that about 25% to 40% of diabetic patients develop DKD, with older diabetic patients being at a significantly higher risk of developing chronic kidney disease (CKD) (2).

**Figure 1:**
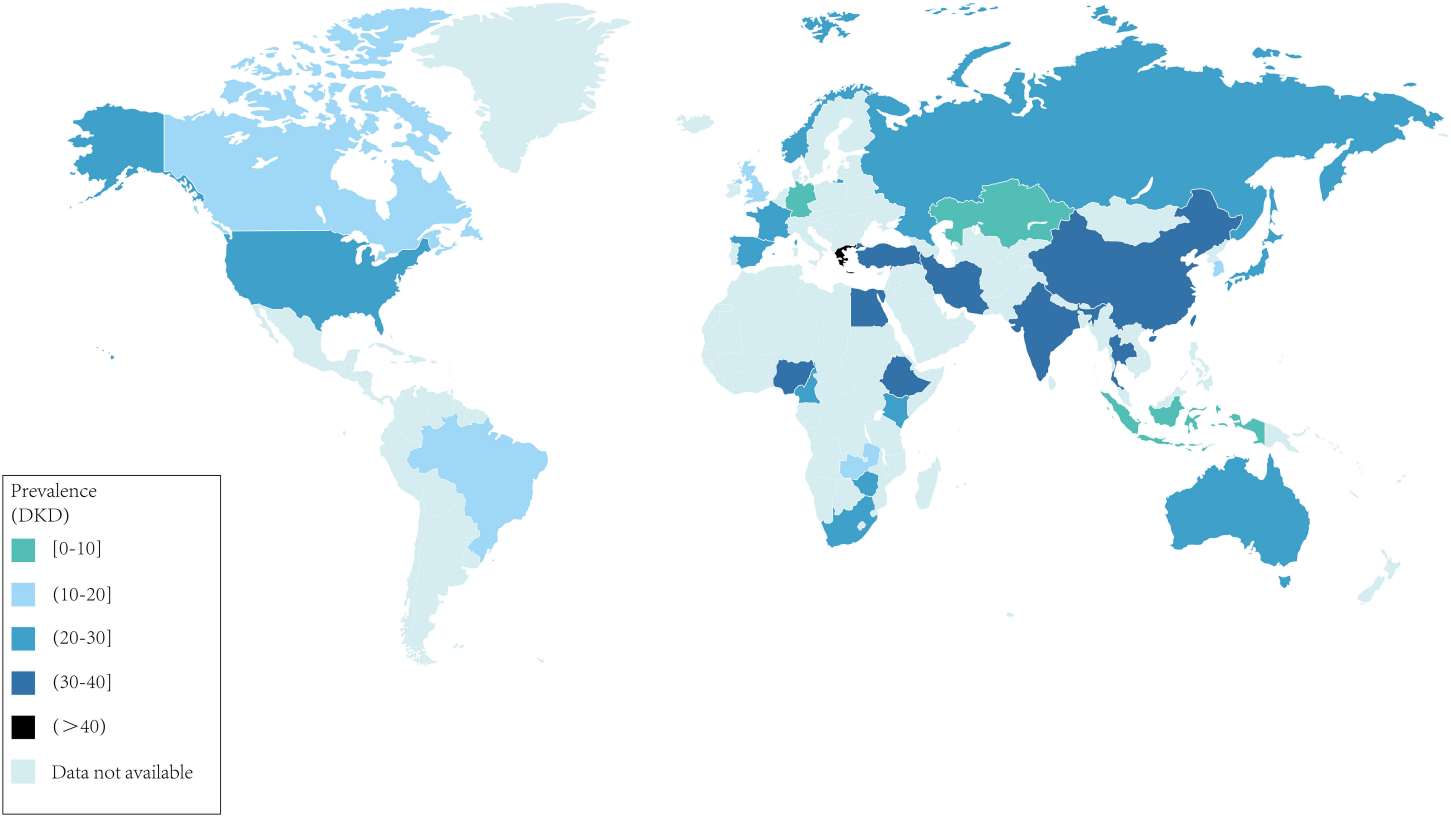
Worldwide prevalence of DKD in patients with diabetes. data are expressed as a percentage of the total population of diabetes patients.

For the diagnosis of DKD, glomerular filtration rate (GFR), elevated urinary albumin excretion (UAE), serum creatinine (Scr), and other relevant indicators in diabetic patients are utilized as diagnostic tools (3). While studies have demonstrated the high sensitivity of the clinical diagnosis of DKD based on changes in biochemical indicators, kidney biopsy continues to play a pivotal role in the diagnosis of DKD, particularly through pathologic examination following renal puncture (4). The prevalence of DKD among diabetic patients across different countries is not well understood. We have summarized this data from our literature survey in **Figure 1** (5–17). Not surprisingly, DKD has become a major public health problem worldwide.

DKD is intricately linked to the metabolic and hemodynamic disorders associated with long-term diabetes mellitus (18). Impaired diabetic microcirculation not only plays a crucial role in the development of diabetic microvascular complications but also contributes to insulin resistance and the progression of diabetes mellitus (19, 20). Persistent metabolic and hemodynamic abnormalities can lead to structural damage in the renal microvasculature, including increased vascular permeability, capillary leakage, thickening of the glomerular basement membrane (GBM), narrowing or even occlusion of the vascular lumen, and microthrombosis, all of which contribute to renal microcirculatory disorders (21).

The focus of this review is to describe the vascular and microcirculatory dysfunctions and their underlying mechanisms in DKD, as well as to summarize the techniques for assessing renal microcirculatory function. Our goal is to provide a comprehensive and up-to-date review that will contribute to the development of safe and reliable therapeutic approaches, thereby advancing the treatment of human DKD.

## 2 Renal microcirculation

### 2.1 Renal microcirculation network

The kidney is a highly vascularized organ. The main renal artery progressively branches into interlobar arteries, arcuate arteries, interlobular arteries, and glomerular afferent arterioles, which ultimately lead to the glomerular capillaries where fluids and solutes are filtered (excluding plasma proteins). Subsequently, the other end of the glomerular capillaries forms the glomerular efferent arterioles, which enter the peritubular capillary network. This network plays a crucial role in filtration, secretion, and reabsorption within the renal tubules, facilitating the removal of waste products from the filtered blood so they can be excreted in the urine. The peritubular capillary network further branches into the arteriolae rectae that extend into the renal medulla. Together with the straight venules, these form the renal medullary microcirculation, which subsequently merges into the interlobular veins, arcuate veins, interlobar veins, and eventually the renal veins (22). The microcirculation is a network of end-vessels composed of microvessels <20 μm in diameter, including small arteries, small veins, and capillaries in between (23). The renal microcirculation is composed of two parts: the renal medullary microcirculation and the renal cortical microcirculation. The renal cortical microcirculation includes the glomerular capillaries and the peritubular capillary network, while the renal medullary microcirculation comprises the arteriolae rectae, straight venules, and the capillaries between them. Glomerular capillaries are composed of endothelial cells, pericytes, and basement membranes, while small arterioles and veins contain an additional layer of smooth muscle cells (24). Functionally, the renal microcirculation is the primary site for the exchange of gases, nutrients, wastes, proteins, and drug components between the blood and tissues. Blood pressure is higher in the glomerular capillaries, where blood gradually passes through the filtration barrier, which is composed of endothelial cells, the basement membrane, and podocytes. At this stage, large amounts of water and solutes are filtered into the renal capsule to form proto-urine. The renal capsule extends outward to form the renal tubules. As the proto-urine enters the tubules, almost all glucose and amino acids, along with most of the water and ions, are reabsorbed into the capillaries surrounding the tubules and then circulate throughout the body with the blood. On the other hand, metabolites reabsorbed by renal tubular epithelial cells (RTECs) enter the primary urine and are excreted along with other waste products. Injuries to the renal microcirculation can be categorized into two types: functional injuries and structural injuries. Functional damage refers to abnormal perfusion without changes in the number or structure of blood vessels. Structural damage involves a decrease in the number of vessels or alterations in their structure. These two types of injuries are not independent of each other and can co-exist. It has been shown that both structural changes in the vasculature and a reduction in the number of capillaries can contribute to the progression of kidney disease (25, 26).

### 2.2 Intrarenal vascular resistance

The microcirculation plays a major role in vascular resistance, and the renal microcirculation can be regulated to maintain glomerular filtration and blood flow. There are three main types of regulation in the renal microcirculation: First, changes in vascular morphology, such as vessel wall hypertrophy, reduce lumen diameter and decrease vasodilatory capacity, leading to increased resistance to blood flow (27); Second, alterations in the kidney’s ability to self-regulate in response to changes in arterial pressure. Renal self-regulation has two components: an intrinsic myogenic response and a glomerular feedback mechanism. When the glomerular filtration rate increases, the rise in sodium chloride flow activates the macula densa and initiates a tubuloglomerular feedback response (28). Third, the effect of vasoactive factors on the renal vasculature. Various pathological conditions result in increased responsiveness of renal microvessels to vasoactive factors, which may lead to changes in renal vascular resistance (29).

**Figure 2:**
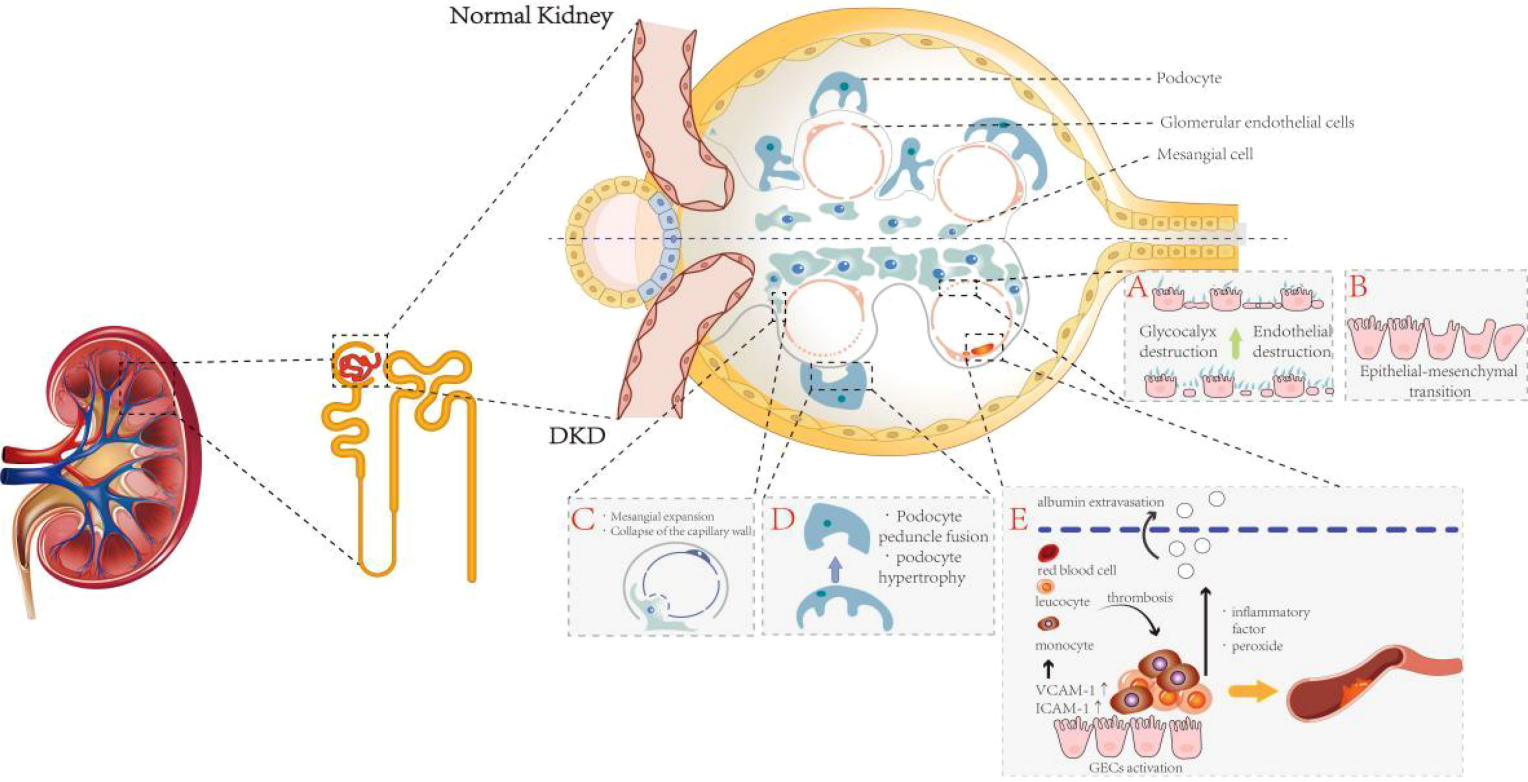
The pathological changes of glomerular cells in DKD. This figure is divided into upper and lower panels to illustrate the differences between normal and DKD glomeruli, with particular emphasis on pathological alterations characteristic of DKD glomeruli: **(A)** Hyperglycemic environments induce degradation of the glycocalyx and injury to GECs. **(B)** GECs undergo endothelial-to-mesenchymal transition. **(C)** MCs proliferate and expand, adhering to the inner layer of the GBM, which triggers capillary detachment and subsequent collapse. **(D)** In the pathological milieu of DKD, podocytes undergo morphological and functional alterations, primarily manifesting as cellular hypertrophy and effacement of foot processes. **(E)** Activated GECs secrete abundant adhesion molecules, recruiting leukocytes and monocytes from the bloodstream to infiltrate the subendothelial layer and form microthrombi, ultimately inducing glomerular microcirculatory dysfunction.

During the progression of DKD due to various pathological factors, the morphology and function of glomerular cells—primarily glomerular endothelial cells (GECs), mesangial cells (MCs), and podocytes—as well as the glomerular basement membrane (GBM), undergo significant changes (**Figure 2**). The damage to these cells leads to pathological changes in renal structure and vasculature, which ultimately manifest as renal hemodynamic alterations, such as increased intrarenal vascular resistance, altered renal blood flow, and elevated renal vascular pressure. Additionally, glomerular hyperfiltration, abnormal renal function, and increased proteinuria are all manifestations of renal hemodynamic abnormalities, indirectly reflecting changes in the renal microcirculation (30).

GECs are located in the innermost layer of the glomerular capillaries, forming the inner wall of blood vessels, and are dynamic regulatory cells that continuously line the entire lumen of these vessels. Therefore, the interaction of GECs with circulating substances is strongly linked to the state of the renal microcirculation (31). GECs serve as the primary barrier maintaining vascular permeability, preventing the leakage of macromolecules from the blood. Additionally, they are the targets of metabolic substances and hemodynamic signals that regulate the glomerular microcirculation (32), playing a crucial role in the occurrence and development of renal microcirculatory dysfunction (33). Because GECs are in direct contact with the blood, they are susceptible to the influence or damage from circulating substances such as glucose, lipids, and inflammatory factors. These cells, in turn, regulate the structure and function of the vasculature through the release of biochemical factors such as nitric oxide and prostacyclin (34). Activated GECs can produce a large number of adhesion molecules, such as vascular cell adhesion molecule-1 (VCAM-1) and intercellular adhesion molecule-1 (ICAM-1), which recruit leukocytes and monocytes from the blood to infiltrate the subendothelial layer and form microthrombi, leading to glomerular microcirculation dysfunction (35, 36). Furthermore, the overexpression of endothelin-1 (ET-1) and angiotensin II (Ang II) induced by a hyperglycemic environment can lead to glomerular endothelial dysfunction, ultimately resulting in malignant nephrosclerosis (37). Additionally, evidence suggests that the dual ET-1 and Ang II receptor antagonistic effects of Sparsentan can improve renal hemodynamics, as well as podocyte and endothelial cell function, in mouse models of focal segmental glomerulosclerosis (38). In addition, activated leukocytes and GECs release inflammatory factors, peroxides, and other factors that damage microvascular endothelial cells and vascular basement membranes, leading to albumin extravasation (39, 40). Thus, the functional state of GECs plays a crucial role in local vasodilation, the maintenance of vascular homeostasis, and selective glomerular filtration.

The maintenance of endothelial cell phenotype is a physiological activity that requires intracellular energy and signaling inputs (41). In pathological states, GECs undergo endothelial-to-mesenchymal transition (EndMT), resulting in reduced glomerular capillary density, loss of glomerular endothelial permeability, and ultimately leading to renal fibrosis and dysfunction (42, 43). In DKD pathology, the loss of fenestrae and the formation of diaphragms induce an increase in GEC permeability and disrupt the ultrastructure of the renal capillary wall. This, in turn, leads to GEC dysfunction and impairs the transport of macromolecules such as albumin through the endothelium (44, 45). The complex meshwork covering the surface of GECs is known as the glycocalyx, located at the interface between endothelial cells and circulating blood. The glycocalyx regulates capillary permeability, limits the adhesion of leukocytes and platelets to GECs, and modulates the transmission of relevant signals. It serves as an important component of the glomerular filtration barrier (GFB) (46, 47). In a high-glucose environment, the production of reactive oxygen species (ROS) and pro-inflammatory cytokines can directly or indirectly lead to the degradation and destruction of the endothelial glycocalyx. This degradation, in turn, triggers endothelial dysfunction, proteinuria, and renal capillary obstruction due to leukocyte deposition (48).

In addition, the reduced concentration of glycosaminoglycans and proteoglycans in the superficial layer of the glomerular capillary lumen in a high-glucose state also leads to dysfunction of the GFB, resulting in albumin leakage, the development of albuminuria, and impaired renal function (49). Under diabetic conditions, the glomerular vasculature is exposed to ischemia, oxidative stress, inflammation, abnormal renin-angiotensin (Ang) secretion, and other injurious factors, leading to necrosis or apoptosis of glomerular vascular endothelial cells and their detachment from the basement membrane. This process results in a decrease in the number of GECs, their dysfunction, and subsequent damage to the GFB (50).

Morphologic changes and dysfunction of glomerular podocytes are also involved in the development of renal microcirculatory disorders and glomerulosclerosis. Glomerular podocytes are highly differentiated, terminally differentiated cells that cover the outer part of the GBM (51). Together with GECs, they are responsible for constituting the GBM, regulating the glomerular filtration rate, and maintaining the shape and integrity of glomerular capillaries. Capillary collaterals are supported by multiple podocytes, and each podocyte simultaneously supports multiple capillary collaterals. To accommodate their physiological functions, podocytes have a unique morphology with a complex cytoskeleton underlying their delicate structure (52). Podocytes possess primary, secondary, and tertiary foot processes, all of which contain a rich actin cytoskeleton. These processes are interspersed and connected by the slit diaphragm (53). Podocyte foot processes regulate glomerular filtration by contracting and expanding, thereby altering the size of the slit diaphragm (52). In the pathological setting of DKD, podocytes undergo morphological and functional changes, including hypertrophy, decreased motility, fusion and loss of foot processes, detachment, autophagy, and apoptosis (54). These complex pathological changes are mediated by various factors such as Ang II, vascular endothelial growth factor (VEGF), reactive oxygen species (ROS), and TGF-β1 (55).

MCs, as stromal cells, are an integral part of glomerular structure and maintain homeostasis of GECs and podocytes (56). In most cases, mesangial cells (MCs) and glomerular endothelial cells (GECs) are tightly coupled, with MCs controlling the surface area of glomerular capillaries by relaxing and contracting, thereby affecting the glomerular filtration rate (GFR) (57). A hyperglycemic environment promotes the proliferation, hypertrophy, and fibrosis of glomerular MCs through various mechanisms, leading to a series of pathological changes, including hemodynamic alterations and neovascularization (58). Hyperglycemia can induce the apoptosis of MCs (59), which in turn leads to impaired glomerular capillary integrity and damage to the glomerular capillary network. This damage can manifest as capillary aneurysms and delayed capillary repair (58). Relatively, mesangial cells (MCs) can partially come into contact with the inner layer of the glomerular basement membrane (GBM) through proliferation and expansion, resulting in capillary collapse as the capillaries become separated from the GBM (60). In addition, due to mesangial expansion, the proportion of MCs in the glomerulus increases, along with an increase in the extracellular matrix (ECM) secreted by MCs and mesangial stroma. Notably, one of the features of glomerulosclerosis is the obstruction of glomerular capillaries by the ECM (61). Thus, lesions of the MCs are closely linked to changes in the renal microcirculation.

The GBM is located between the GECs and the podocytes. The GBM is a crucial component of the GFB and also provides structural support to glomerular cells (62). GBM thickening is one of the earliest and most characteristic changes in diabetic glomeruli (40, 63, 64). Thickening and sclerosis of the GBM may decrease the elasticity of the capillary wall and promote glomerular injury through hemodynamic mechanisms (65). Additionally, the thickened GBM can adhere to the renal capsule, further promoting glomerulosclerosis (55). It has been demonstrated that GBM thickening is positively correlated with UACR and negatively correlated with eGFR (39).

Under the conditions of DKD, GECs, podocytes, and MCs undergo abnormal structural and functional changes, engaging in crosstalk with each other. These interactions result in the unique structural features and microcirculatory abnormalities characteristic of DKD kidneys (66). However, the manifestations of microcirculatory abnormalities in DKD are highly diverse, and the associated mechanisms are extremely complex. In this review, we focus on the alterations in renal microvascular structure, microcirculatory hemodynamic abnormalities, and glomerular vascular endothelial cell dysfunction in DKD, with the aim of providing new insights for the study of this condition.

## 3 Mechanism of microcirculation dysfunction

### 3.1 Nitric oxide

NO is an endogenous vasodilatory molecule and an anti-inflammatory signaling molecule that maintains normal renal vascular resistance and vascular homeostasis. It plays a crucial regulatory role in the maintenance of renal hemodynamics and glomerular function (67). NO is synthesized by endothelial nitric oxide synthase (eNOS) and neuronal nitric oxide synthase(nNOS) in vascular endothelial cells in response to stimuli such as hypoxia and shear stress (68, 69). The released NO mediates local vasodilation, antagonizes platelet aggregation, and inhibits vascular smooth muscle cell proliferation (70). On the other hand, the expression of eNOS is primarily localized in the endothelium of preglomerular and postglomerular vessels, as well as in glomerular capillaries. In the early stages of DKD, increased eNOS phosphorylation and a surge in NO production may contribute to glomerular hyperfiltration (71). It has been demonstrated that upregulation of eNOS in the microcirculatory system of rats with early diabetic nephropathy promotes NO production and leads to the dilation of renal microvessels (72). Genetic polymorphisms in eNOS as a factor contributing to the worsening of DKD (73). As DKD progresses, NO bioavailability decreases and NO synthesis is disrupted due to eNOS uncoupling, leading to hypertension and renal vasoconstriction (74, 75). An animal experiment confirmed this: Diabetic eNOS knockout mice exhibited clear microangiopathy due to defects in NO production. Significant microaneurysms, mesangial expansion, capillary endothelial damage, and occasional arteriolar hyalinosis were observed (76). With the progression of diabetes, advanced glycation end-products (AGEs) gradually accumulate. A study has shown that excessive AGEs significantly reduce eNOS activity and cellular NO levels, thereby causing endothelial dysfunction (77). High levels of reactive oxygen species (ROS) can lead to oxidative modifications of proteins, loss of enzyme activity, alterations in cellular function, and disruption of cellular homeostasis (78). The relationship between NO and ROS is bidirectional. Low levels of NO in endothelial cells induce the expression of antioxidant genes, whereas elevated ROS levels suppress NO production by inhibiting NOS (79). In the kidney, elevated ROS levels lead to a deficiency of NO and NOS, resulting in renal endothelial dysfunction, increased vascular resistance, and reduced vasodilation (77, 80, 81). In another study, it was shown that endothelium-derived NO plays a role in counterbalancing the vasoconstrictive effects of Ang II (82). eNOS knockout mice were significantly more responsive to Ang II (82). Another experiment demonstrated that the absence of eNOS has a more detrimental effect on the renal microvascular system than on aortic blood vessels. In a high-glucose environment lacking eNOS, mice exhibited significant thickening of the GBM and developed pronounced albuminuria (83). The researchers also observed fibrin, platelets, and leukocytes accumulating in the kidney capillaries, similar to the characteristics of glomerular injury seen in thrombotic microangiopathy. Additionally, NO deficiency in diabetes causes VEGFA to become harmful to glomerular cells, leading to abnormal vascular repair and remodeling (82, 84).

### 3.2 Endothelin-1

ET-1 is an endothelium-derived polypeptide with potent vasoconstrictive effects, playing a key role in circulatory homeostasis (85). ET-1 induces a range of pathophysiological changes by binding to the ETA receptor (ETAR) and ETB receptor (ETBR) (86). Activation of ETAR can lead to responses such as vasoconstriction and inflammation, while activation of ETBR mediates vasodilation (86). ETAR is predominantly found on renal vasculature, MCs, and podocytes, whereas ETBR is primarily located on renal tubules. The upregulation of ET-1 secretion can result in diverse effects due to the widespread presence of endothelin receptors within glomerular structures (86). It has been demonstrated that under conditions of diabetes mellitus, ET-1 concentrations show a marked elevation in both humans and experimental animals (87, 88). ET-1 mediates the effects of IL-15 or directly activates endothelin receptors in podocytes, leading to podocyte damage and glomerulosclerosis (89, 90). In addition, ET-1 can induce the release of heparinase from podocytes, thereby destroying the endothelial glycocalyx—a process that is exacerbated in diabetes (91). In a mouse model of DKD, researchers found that glomerular endothelial mitochondrial dysfunction was associated with increased expression of glomerular ETAR and circulating ET-1. Blockade of ETAR prevented mitochondrial oxidative stress in GECs, which ameliorated endothelial injury, podocyte loss, albuminuria, and glomerulosclerosis (92). In DKD, ET-1 also affects MCs, and the binding of ET-1 to ETAR in MCs promotes the RhoA/ROCK pathway, accelerating MC proliferation and ECM accumulation, which can impact glomerular capillary homeostasis (93). In terms of hemodynamics, ET-1 levels are upregulated under hyperglycemic conditions, causing constriction of the renal vasculature and a decrease in renal blood flow, which subsequently leads to a reduction in glomerular filtration rate (94). Interestingly, afferent arterioles appear to be more sensitive to the effects of ET-1 (95). In a study measuring plasma ET-1 concentrations in 99 diabetic patients, researchers found that plasma ET-1 levels were significantly higher in diabetic patients compared to normal subjects and that ET-1 concentrations were inversely correlated with effective renal plasma flow, demonstrating the potential negative impact of ET-1 on renal circulation (96). In animal experiments, intravenous administration of exogenous ET-1 caused a slight increase in blood pressure and a significant reduction in renal cortical and medullary blood flow in rats (97). In another study, researchers found that DKD patients had higher levels of ET-1 expression in kidney capillaries compared to normal subjects, as determined by staining, suggesting a potential effect of ET-1 on kidney microvessels (98). In summary, aberrant expression of ET-1 in DKD is a significant cause of reduced renal blood flow and impaired renal microcirculation. Moreover, studies have shown that ET-1 also affects podocytes. Podocytes are highly differentiated cells with a complex actin cytoskeleton (99). In podocytes, ET-1 can act through both paracrine and autocrine mechanisms. The increase in ET-1 induces the redistribution of actin fibers toward the cell periphery and promotes foot process effacement (100). On the other hand, under ET-1 stimulation, the ETAR forms a complex with the scaffold proteinβ-arrestin-1 and the tyrosine kinase Src. This complex subsequently transactivates the EGFR, phosphorylates β-catenin, and promotes the transcription of mesenchymal markers. This molecular cascade leads to a migratory phenotype in podocytes, enhancing their detachment from the GBM (101). ET-1 can also serve as a mediator of crosstalk between GECs and podocytes, contributing to the progression of DKD. In DKD, the activation of TGF-β receptors increases the expression of ET-1 in podocytes. ET-1 acts via a paracrine mechanism to stimulate ETAR in adjacent GECs. The activation of these ETAR leads to mitochondrial stress and oxidative stress in endothelial cells (102). Another study demonstrated that podocyte-derived ET-1 increases heparanase and hyaluronidase levels in GECs, leading to damage to the endothelial glycocalyx, glomerular endothelial injury, and albuminuria. Inhibition of the type A endothelin receptor, rather than the type B endothelin receptor, reduces endothelial injury (103).

### 3.3 Polyol pathway

The polyol pathway is a form of glucose metabolism that is significantly activated in response to high intracellular glucose concentrations. Under normal conditions, cells produce pyruvate by using glucose as an energy source through phosphorylation in the presence of hexokinase. However, when intracellular glucose concentrations become excessive, hexokinase becomes saturated, allowing more glucose to enter the polyol pathway (104). Nicotinamide adenine dinucleotide phosphate hydrogen (NADPH) plays an important role in the polyol pathway reactions (104). Glucose is converted to sorbitol by aldose reductase, with NADPH acting as a coenzyme in this process. Subsequently, sorbitol is converted to fructose by sorbitol dehydrogenase, where NAD is involved, producing NADH (105). Increased intracellular sorbitol leads to decreased Na+-K+ ATPase activity at the cell membrane and inhibits the entry of myo-inositol into the cell (106). Thus, activation of the polyol pathway in DKD causing an increase in sorbitol and fructose concentrations and a decrease in intracellular myo-inositol levels. This disruption in cellular osmoregulation promotes the development of diabetic microvascular complications. An animal experiment showed that elevated concentrations of sorbitol in a mouse model of renal aldose reductase overexpression led to renal vascular thrombosis and fibrinous deposits in Bowman’s capsule (107). In another study, glomerular hypertension, vasoconstriction of the renal cortex, and thickening of the vascular walls of afferent arterioles were observed in rats fed a high-fructose diet compared to those on a normal diet. These lesions may be associated with uric acid produced by fructose metabolism (108). In addition, reduced glutathione (GSH) acts as a scavenger of reactive oxygen species (ROS). As a cofactor for GSH, the depletion of NADPH in the polyol pathway may induce or exacerbate oxidative stress in cells, leading to kidney damage (109). Activation of the polyol pathway occurs not only at elevated glucose concentrations but also during the secondary depletion of NADPH, which shifts glucose metabolism from glycolysis to other pathways. This shift induces the activation of the renin-angiotensin-aldosterone system (RAAS) and leads to renal injury (110).

### 3.4 Advanced glycosylation end products

AGEs are stable covalent adducts formed by the spontaneous reaction of macromolecules such as proteins, lipids, or nucleic acids with glucose or other reducing monosaccharides, without the involvement of enzymes (111). AGEs accumulate gradually with metabolism and age. In diabetic patients, the synthesis and accumulation of AGEs are accelerated, and even after hyperglycemia is corrected, AGEs levels do not return to normal but persist in the blood vessels due to slow degradation over time (112). AGEs are primarily expressed on renal tubules, GECs, and MCs. AGEs bind to the receptor for advanced glycation end-products (RAGE) and activate a series of signaling pathways, triggering adverse effects such as cell proliferation, inflammatory responses, and apoptosis (113). It has been shown that in human microvascular endothelial cells (HMVECs), AGEs bind to RAGE, leading to the upregulation of heparinase expression through the activation of FOXO4 (114). This upregulation may be related to FOXO4-mediated oxidative stress (115), which is potentially one of the mechanisms underlying microvascular complications in diabetes mellitus. In another study, a two-part *in vivo* and *in vitro* experiment demonstrated that the binding of AGEs to RAGE activated the RAGE/RhoA/ROCK signaling pathway and upregulated the expression of VEGF, MCP-1, and ICAM-1. This upregulation led to macrophage infiltration and glomerular endothelial dysfunction, resulting in impaired renal microcirculation (116). In the kidney, RAGE signaling activates the transcription factor NF-κB, promoting the release of cytokines and tissue factors while reducing NO release (117). Additionally, the AGEs/RAGE axis inhibits the eNOS activity of endothelial cells, contributing to the development of DKD (118). RAGE knockout mice have also been shown to be protected from various features of DKD, such as reduced glomerular filtration rate, albuminuria, and glomerulosclerosis (119).

### 3.5 VEGF

VEGF is a member of a protein family that includes VEGF-B, VEGF-C, VEGF-D, VEGF-E, and placental growth factor (PlGF). Given the predominant role of VEGF-A in regulating angiogenesis and vascular function, it is often referred to simply as VEGF and plays a crucial role in the development of DKD (120). There are three types of VEGF receptors: VEGF receptor 1 (R1), VEGF receptor 2 (R2), and VEGF receptor 3 (R3). R1 and R2 are primarily expressed on endothelial cells. According to the study, VEGF-A binds to R1 and R2, VEGF-B and PlGF bind to R1, and VEGF-C and VEGF-D bind to R3, but can also bind to R2 after hydrolytic cleavage (121). It has been suggested that typical intraglomerular VEGF signaling primarily involves the binding of VEGF-A to R2, which is expressed by glomerular endothelial cells (122). VEGF plays a crucial role in the development of DKD, and the integrity of the vascular system relies on a balance between various vascular factors (123). Disruption of this balance has been observed in several kidney diseases, particularly DKD (124). VEGF-A is essential for the proliferation, differentiation, and survival of endothelial cells in the vascular system, and thus it plays a significant role in regulating endothelium-dependent vasodilation and vascular permeability (125). Over the past two decades, studies of kidney disease have shown that dysregulated VEGF-A expression plays a critical role in damaging the renal capillary network. Low expression of VEGF-A promotes renal microvascular thrombosis, while high expression of VEGF-A leads to proteinuria (126, 127). In the early stage of DKD, VEGF-A expression is increased in glomerular podocytes (128), and the over-secreted VEGF-A crosses the glomerular basement membrane, binds to and promotes the dimerization of R2 expressed on the surface of glomerular endothelial cells. This binding leads to the phosphorylation of R2’s structural domains and the activation of downstream signaling pathways that promote capillary sprouting in the kidney (129). In addition, elevated VEGF-A expression can promote macrophage migration and infiltration in the glomerulus, leading to pathological changes in the renal microcirculation, such as increased renal vascular permeability, glomerular inflammation, and glomerular microaneurysm formation (130). Interestingly, in the early stages of DKD, VEGF-C appears to have a protective effect on the kidney. One study suggests that VEGF-C may inhibit the VEGF-A signaling pathway by competing with VEGF-A for binding to R1 and R2, thereby exerting its protective effect (131). As DKD progresses, reduced VEGF-A levels lead to the thinning of glomerular capillaries, possibly due to a decrease in podocytes (122). A study demonstrated that glomerular VEGF-A mRNA levels were 2.5 times lower in patients with progressive DKD than in normal subjects (68). Furthermore, another study demonstrated that in endothelial-specific Dgkϵ knockout mice, the loss of Dgkϵ in endothelial cells impairs downstream VEGFR2 signaling, preventing the activation of Akt. This results in defective induction of COX-2 and prostaglandin E2 (PGE2), ultimately leading to thrombotic microangiopathy and proteinuria in mice (132). These studies suggest that maintaining podocyte-derived VEGF-A expression within the normal range is essential for preserving the structure and function of renal capillaries (70).

### 3.6 The renin angiotensin aldosterone system

The development of several renal diseases is associated with the activation of the RAAS, and in DKD, RAAS plays a key role. The RAAS regulates renal vasoconstriction and vasodilation, maintains electrolyte homeostasis, and modulates renal tissue growth. However, under pathological conditions, abnormal activation of the RAAS can lead to the constriction of small renal arterioles and glomerular capillaries, resulting in increased peripheral and renal microcirculatory resistance, and inducing vascular endothelial dysfunction (133). A study suggests that RAAS is abnormally activated in a high-glucose environment, stimulating the expression of renin and Angiotensin (Ang) in the kidney. Hyperactivation of endogenous renal RAAS appears to affect afferent arterioles more than efferent arterioles. One study found that in DKD due to type 1 diabetic disease, afferent arterioles show more pronounced constriction and increased vascular resistance (134). However, in most cases, Ang II promotes renal tissue fibrosis, vasoconstriction of the efferent arteriole, increased intraglomerular pressure, a decreased number of small blood vessels, and other pathological changes (135, 136). In diabetic rats, upregulation of Ang II can damage the glomerular filtration barrier and increase glomerular permeability, leading to proteinuria. Pathohistological changes, such as mesangial expansion, diffuse thickening of the basement membrane, and effacement of podocyte foot processes, have also been observed (137). At the same time, increased Ang II can stimulate the synthesis of renal ET-1, thereby inducing kidney injury through an additional pathway. In diabetes mellitus, increased binding of aldosterone to the mineralocorticoid receptor (MR) further contributes to renal fibrosis (138). Activation of the mineralocorticoid receptor (MR) can also lead to reduced eNOS activity, thereby affecting vasoconstriction and vasodilation (139). Several studies have shown that treatment with MR antagonists significantly improves the urinary albumin-to-creatinine ratio and reduces the risk of cardiovascular events in patients with DKD (140, 141). This ameliorative effect was similarly demonstrated in animal studies, showing a reduction in renal fibrosis, protection of glomerular structure, and improvement in podocytopathy (142). However, the elevation of blood potassium associated with aldosterone receptor antagonism is a concern.

### 3.7 ROS

ROS are key mediators of glomerular cell injury in diabetes. Oxidative stress occurs when ROS production exceeds their scavenging by antioxidants, initiating and mediating the signaling cascade in response to cell injury. A growing body of research suggests that overproduction of ROS is a critical factor linking altered renal metabolic pathways to the hemodynamic disturbances of DKD. These pathways ultimately lead to inflammation, fibrosis, and endothelial dysfunction (142, 143). In the early stages of DKD, mitochondrial dysfunction occurs in both glomerular podocytes and (RTECs). This dysfunction leads to enhanced mitochondrial substrate oxidation, resulting in the overproduction of ROS and subsequent oxidative stress (144). Multiple enzyme systems associated with ROS production are present in the kidney, with the NADPH oxidase (NOX) family being the most significant contributors (74, 75). The expression of NOXs is upregulated in a hyperglycemic environment. Studies have shown that in the kidneys of NOX2-overexpressing mice, peroxide levels are significantly increased, glomerular endothelial cells are activated, and the endothelial glycocalyx is reduced (145). In addition, ROS are also produced during uric acid production. Hypoxanthine is oxidized to xanthine by xanthine oxidase (XO), and xanthine is further oxidized to uric acid by the same enzyme, producing both O_2_ and H_2_O_2_ in the process (146). Upregulation of Ang II and adenosine activates NOX in the renal microvasculature via AT1R and A1R, leading to superoxide production (147). Oxidative stress in the kidney contributes to renal vascular remodeling, while increased ROS prompts vasoconstriction of renal afferent arterioles, enhances myogenic responses, and alters tubuloglomerular feedback (TGF), further contributing to renal hemodynamic dysfunction in DKD (147). It has been demonstrated that the overproduction of H2O2 in a CKD mouse model leads to myogenic constriction of renal arteries in mice (148). Additionally, ROS have been shown to increase the responsiveness of renal afferent arterioles to Ang II, leading to their constriction and inhibiting the production of NO (149). Another experiment showed that in a diabetic mouse model, the high-glucose environment led to the activation of Wnt signaling and promoted ROS production, particularly H_2_O_2_. The increased ROS enhanced the responsiveness of renal afferent arterioles to ET-1, leading to their constriction. Additionally, blocking the Wnt signaling pathway increased catalase concentration in mice, which corrected the vascular abnormalities in DKD (150). On the therapeutic side, one study found that acetylcholine-induced endothelium-dependent relaxation of renal afferent arterioles was lower in diabetic mice compared to normal mice, due to a reduction in NO and the overproduction of ROS. This finding suggests a potential inhibitory effect of ROS on renal vasodilation. Meanwhile, the researchers discovered that fenofibrate enhances renal vasorelaxation and improves renal microcirculation by activating the PPAR/LKB1/AMPK/eNOS pathway, promoting endogenous NO production, and preventing oxidative stress (151).

### 3.8 Inflammasome

The inflammasome is a multiprotein complex critical to the immune system, responsible for detecting and responding to pathogenic microorganisms and cellular stimuli, thereby activating the inflammatory response. These stimuli include pathogen-associated molecular patterns (PAMPs) such as bacterial lipopolysaccharide (LPS) and viral RNA, as well as damage-associated molecular patterns (DAMPs) like ATP and β-amyloid protein (152). In DKD, the NLRP3 inflammasome is a key mediator of inflammation associated with disease progression. Under DKD conditions, various DAMPs can activate the NLRP3 inflammasome. AGEs formed under hyperglycemic conditions, mitochondrial dysfunction, and increased ROS production all contribute to and exacerbate NLRP3 activation. The activation of NLRP3 involves two steps: priming and activation. The priming step is initiated through the recognition of PAMPs or DAMPs (153, 154). This recognition triggers signaling pathways, including MAPK and NF-κB, leading to the upregulation of NLRP3. The activation step occurs in response to the priming signal, which induces the assembly of the NLRP3 inflammasome complex, where NLRP3 recruits the adaptor protein ASC and pro-caspase-1 (155). The formation of this complex leads to the activation of caspase-1, which subsequently cleaves pro-IL-1β and pro-IL-18 into their active forms, IL-1β and IL-18. These cytokines are then released to mediate the inflammatory response (156).

Studies have shown that pharmacological inhibition of caspase-1 or NLRP3 knockdown reduces inflammasome activation and thrombosis under hypoxic conditions. Furthermore, the early pro-inflammatory state induced by HIF-1α-activated NLRP3 inflammasomes in venous settings is a key factor in acute thrombotic events under hypoxic conditions (157). In addition, activation of the NLRP3 inflammasome can exacerbate the calcification of vascular smooth muscle cells (158), further demonstrating the potential impact of NLRP3 inflammasomes on the microcirculatory system. The accumulation of inflammatory cells in the kidney is closely associated with decreased renal function (159, 160). Inflammasome activation plays a significant role in the pathology of DKD, and NLRP3 can be activated by metabolites associated with DKD, such as AGEs and ROS (161, 162). Activation of the NLRP3 inflammasome leads to elevated levels of IL-1ß and IL-8 in DKD patients (163, 164). In patients with CKD, the expression of inflammasome activation markers (CASP1, IL-1β, and IL-18) in renal biopsy samples is positively correlated with the severity of albuminuria (161). Similarly, the expression of glomerular inflammasome markers, such as NLRP3, ASC, CASP1, and IL-18, is significantly increased in DKD patients compared to non-diabetic healthy individuals. These inflammasome-associated proteins are also upregulated in the kidneys of db/db mice (162). Activation of the podocyte NLRP3 inflammasome leads to glomerular injury, proteinuria, glomerular mesangial expansion, and glomerular basement membrane thickening. It also exerts immune cell-like functions that exacerbate renal microcirculatory disturbances in DKD (165). Not surprisingly, inhibition of high glucose-induced NLRP3 inflammasome activation in podocytes attenuated podocyte injury (166). Activation of NLRP3 can also lead to glomerular endothelial dysfunction. Studies have shown that biomarkers of neutrophil extracellular traps (NETs) are increased in both diabetic mice and diabetic patients. In cellular experiments, it was demonstrated that while a high-glucose environment induced IL-1β and NLRP3 in glomerular endothelial cells (GECs), NETs further exacerbated NLRP3 activation, thereby contributing to NLRP3-induced glomerular endothelial dysfunction (165). In addition, NLRP3 inflammasome activation can mediate Ang II-induced podocyte apoptosis and mitochondrial dysfunction, exacerbating renal microcirculatory injury and thereby promoting proteinuria and glomerulosclerosis in DKD patients (167, 168). Notably, Gasdermins (GSDMs) are pore-forming proteins that execute pyroptosis. They are activated via canonical inflammasomes, noncanonical pathways, or other triggers, enabling membrane pore formation to induce pyroptosis and subsequent release of inflammatory mediators (169). In the canonical inflammasome pathway, caspase-1 activation downstream of inflammasome assembly cleaves gasdermin D (GSDMD), generating its N-terminal fragment (GSDMD-NT). This fragment oligomerizes to form plasma membrane pores, facilitating pyroptotic cell death and the release of interleukin-1β (IL-1β) and IL-18. In contrast, the non-canonical pathway involves direct activation of caspase-4/5/8/11 by cytosolic lipopolysaccharide (LPS) or bacterial toxins, leading to the same cascaded reactions (169). Targeting GSDMD has emerged as a critical therapeutic strategy in DKD. Studies demonstrate that dapagliflozin significantly reduces renal expression of NLRP3, Caspase-1, and GSDMD-NT in DKD models, suppressing pyroptosis-associated inflammatory responses. Molecular docking assays confirm that dapagliflozin directly binds to GSDMD, blocking its activation (170). Similarly, Astragaloside IV (AS-IV) ameliorates renal function and podocyte injury in db/db mice by inhibiting the TXNIP-NLRP3-GSDMD axis, exerting anti-pyroptotic effects and attenuating DKD progression (171). Additionally, Loganin suppresses the canonical NLRP3/Caspase-1/Gasdermin D pathway, reducing fasting blood glucose, blood urea nitrogen, and serum creatinine levels in DKD mice while alleviating renal pathological changes (172).

The NLRP1 inflammasome is activated by hyperglycemia-associated DAMPs, triggering caspase-1 autocleavage and subsequent release of IL-1β/IL-18, which drives inflammatory responses. Hyperglycemia-induced endoplasmic reticulum (ER) stress further elevates NLRP1 levels via ATF-4 activation, leading to the activation of MAPK, NF-κB, and TGF-β/Smad signaling pathways, thereby promoting fibrogenesis and tissue injury (173). Intriguingly, studies indicate that NLRP1 gain-of-function variants suppress excessive inflammation, suggesting its dual regulatory role in both amplifying inflammatory cascades and maintaining metabolic homeostasis (174).

NLRC4 interacts with the NLR family apoptosis inhibitory protein (NAIP) to form a complex, recruiting apoptosis-associated speck-like protein containing a CARD (ASC) and activating caspase-1, thereby promoting the maturation and release of IL-1β and IL-18 to induce pyroptosis (175). In DKD, hyperglycemic conditions and oxidative stress upregulate and activate NLRC4, triggering IL-1β release and promoting macrophage infiltration in renal tissues. Concurrently, activation of the NF-κB and JNK pathways exacerbates inflammatory responses, elevating pro-fibrotic factors such as TNF-α and TGF-β, ultimately leading to podocyte injury, GBM thickening, and mesangial matrix expansion (176). Studies reveal increased Tim-3 expression in DKD (177), while under renal ischemia-reperfusion injury (IRI), Tim-3 exacerbates kidney damage by upregulating NLRC4 activity, amplifying IL-1β/IL-18-mediated local inflammation. These findings highlight the potential role of NLRC4 in driving DKD progression. An interesting research suggests that: moderate intensity continuous training(MICT) improved renal fibrosis and renal injury, attenuating the inflammatory response by inhibiting TLR4/NF-κB pathway and the activation of NLRC4 inflammasome (178).

Absent in melanoma 2 (AIM2) is expressed in the kidney and predominantly activated by macrophages. Immunofluorescence staining in renal tissues of CKD patients demonstrates AIM2 expression in glomeruli and tubules. *In vitro* studies confirm that macrophages phagocytosing necrotic cells activate caspase-1 and IL-1β through AIM2-dependent mechanisms, driving a pro-inflammatory phenotype and exacerbating chronic kidney injury (179). In DKD, HG conditions induce excessive ROS generation, leading to DNA damage in RTECs, which subsequently activates AIM2. AIM2 facilitates inflammasome assembly by recruiting the adaptor protein apoptosis-associated speck-like protein containing a CARD (ASC) and caspase-1, thereby promoting caspase-1 autocatalytic cleavage. The activated caspase-1 cleaves GSDMD to generate its N-terminal fragment, which forms pores in the cell membrane, triggering pyroptosis and mediating renal tubular epithelial cell injury. Furthermore, elevated AIM2 expression in renal RTECs of DKD patients and db/db mice exhibits a positive correlation with serum creatinine levels and an inverse correlation with eGFR, underscoring the critical association between AIM2 expression and impaired renal function (180). In therapeutic research, a study demonstrated that wogonin mitigates renal inflammation and fibrosis in DKD by upregulating suppressor of cytokine signaling 3 (SOCS3). This upregulation suppresses HG-induced activation of Toll-like receptor 4 (TLR4) and its downstream JAK/STAT signaling pathway, thereby downregulating the AIM2 inflammasome and the expression of associated pro-inflammatory cytokines (181).

NLRP6, also known as NALP6 or PYPAF5, is predominantly expressed in the human and mouse intestine, and to a lesser extent in the liver, brain, kidney, and lungs (182). Previous research demonstrated that co-expression of human NLRP6 and ASC in HEK293T or COS-7 cells triggers caspase-1 activation and subsequent IL-1β secretion, suggesting the potential formation of a functional NLRP6 inflammasome complex (183). Emerging evidence suggests a nephroprotective role of NLRP6 in kidney diseases. Under physiological conditions, NLRP6 maintains cellular homeostasis in renal RTECs by suppressing phosphorylation of ERK1/2 and p38 MAP kinases. However, during acute kidney injury (AKI), NLRP6 expression is markedly downregulated, leading to aberrant activation of MAPK signaling pathways. This dysregulation exacerbates inflammatory responses and promotes tubular cell apoptosis. Notably, a parallel study demonstrated that Nlrp6-deficient mice exhibited exacerbated renal inflammatory responses and fibrotic progression (184). Mechanistically, this phenomenon may arise from Nlrp6 downregulation-triggered activation of the p38 MAPK signaling pathway, which drives upregulation of TGF-β1 and connective tissue growth factor (CTGF) while concurrently suppressing the antifibrotic factor Klotho (185).

### 3.9 Protein kinase C

The PKC family acts as a signaling kinase involved in multiple signaling pathways, including cell proliferation, differentiation, cell cycle regulation, and apoptosis. PKC activation induced by a hyperglycemic environment plays a crucial role in the development of DKD (186). It has been demonstrated that Ang II- and ET-induced renal vasoconstriction is mediated by PKC activation (187). Under conditions of hyperglycemia or insulin resistance, PKC activation in vascular tissues inhibits PI-3 kinase-mediated eNOS expression, leading to endothelial dysfunction, which may contribute to impaired renal microcirculation in DKD (188). Additionally, PKC activation by high glucose concentrations leads to increased expression of VPF-mRNA in VSM cells, which subsequently induces abnormal endothelial permeability and angiogenesis in diabetes mellitus (189).In cellular experiments, PKC activation induced by high glucose concentrations promoted TGF-β1 expression in mesangial cells (MCs), which subsequently led to the accumulation of microvascular matrix proteins (190).

### 3.10 Epidermal growth factor receptor

EGFR is a member of the ErbB/HER family of receptor tyrosine kinases. It has been demonstrated that the deterioration of EGFR tyrosine kinase phosphorylation is a significant factor contributing to diabetic microvascular dysfunction. Treatment of type 2 diabetic mice with an EGFR tyrosine kinase inhibitor for two weeks resulted in a significant improvement in vasotension and endothelial function in mesenteric and coronary arteries (191). In the kidney, EGFR is expressed in various glomerular cells, including podocytes, GECs, and MCs (192). Moreover, EGFR expression is clearly upregulated in a high-glucose environment (193). Studies have shown that antagonizing EGFR improves renal vascular endothelial dysfunction as well as renal hemodynamics (194). Phosphorylation of EGFR mediates vascular remodeling of resistance arteries and increases vessel stiffness and wall thickness in diabetic mouse models (195). These abnormal changes may be mediated through the ERK1/2-ROCK signaling pathway (196). At the cellular level, EGFR activation is involved in the loss of podocytes, tubular cell apoptosis, and glomerulosclerosis in DKD (193). On the other hand, there is a potential link between EGFR and other injury mediators. Studies have demonstrated that ET-1 activates the ETAR, driving podocyte migration through β-arrestin signaling and increasing β-arrestin-1 expression. β-arrestin-1 forms a trimeric complex with Src, leading to EGFR transactivation and β-catenin phosphorylation, which subsequently promotes the gene transcription of Snail. This process results in podocyte loss and the formation of proliferative lesions (101). An animal study demonstrated that targeted knockout of EGFR prevented high-fat diet (HFD)-induced endothelial dysfunction. HFD-induced albuminuria was less pronounced in animals with endothelial EGFR knockout, while it was completely abolished in animals with vascular smooth muscle EGFR knockout. These findings highlight the potential association between EGFR and renal circulatory disorders (197).

In summary, targeted inhibition of EGFR expression may be a potential treatment for DKD.

### 3.11 Platelet-derived growth factors

PDGF is a peptide regulator that stimulates cell growth and is stored in platelet α-granules under physiological conditions, where it is activated and released during blood coagulation. The PDGF family consists of four members: PDGFA, PDGFB, PDGFC, and PDGFD. These four polypeptide units can form five types of dimers: PDGF-AA, PDGF-BB, PDGF-CC, PDGF-DD, and PDGF-AB (198). The five PDGF dimer subtypes exert various biological functions by binding to two specific receptors, PDGFR-α and PDGFR-β (199). PDGF is produced and secreted by various cell types, which promote mitosis and induce the division, proliferation, and migration of vascular cells, this highlights the potential role of PDGF in regulating vascular homeostasis (200).

It has been demonstrated that PDGF induces hypertrophy of vascular smooth muscle cells in the rat kidney, thereby affecting renal perfusion (201). In DKD, PDGF and its receptors are overexpressed (202); PDGF-A is primarily distributed in glomerular and RTECs, while PDGF-B is mainly located extracellularly (202). With the progression of DKD, gradual fibrosis of the kidneys occurs, and one study showed that PDGF-CC plays an important role in the process of renal fibrosis, but does not significantly aggravate capillary rarefaction (203). At the cellular level, PDGF-BB, PDGF-CC, and PDGF-DD all stimulate the proliferation of MCs, leading to mesangial expansion (204). Furthermore, in proximal tubular epithelial cells, increased phosphorylation of PDGF receptor-β (PDGFRβ) due to high glucose activates the Akt/mTORC1 signaling pathway, which in turn promotes the expression of collagen I(α2), ultimately inducing tubulointerstitial fibrosis in DKD (205). In summary, changes in PDGF and its receptors have the potential to influence renal microcirculation in DKD.

## 4 DKD preclinical models

To explore new treatments for DKD and test the effectiveness of therapeutic regimens, it is necessary to develop animal models of DKD with varying characteristics. These models can mimic the human state of DKD, thereby providing valuable clinical evidence for treatment strategies. The most common methods for modeling DKD include the use of chemicals, genetic engineering, genetic hybridization, dietary interventions, or combinations of these approaches (206). Because the symptoms and micropathologic changes of DKD vary among experimental models, it is important to select the appropriate animal model based on the specific research focus (206). Mice are currently the most common animal model for studying DKD because they are inexpensive to maintain, reproduce rapidly, and can reflect the disease progression of DKD to some extent (207). However, mouse models also have disadvantages, including a lack of genetic diversity and differences in islet cell distribution compared to humans (208). Large animal models of DKD, such as diabetic dogs, exhibit characteristics of the disease that are similar to those in humans. However, these models are not widely used in research due to the disadvantages of longer study cycles and higher maintenance costs (209). Here, we summarize the common animal models of DKD (**Table 1**). These models exhibit many of the features of human DKD and the associated changes in renal microcirculation. In the following section, we outline the mechanisms underlying the establishment of commonly used animal models for DKD.

**Table 1:** Pathological manifestations and renal microcirculatory characteristics in diabetic kidney disease animal models.

Model name	Strain	Type of diabetes	Metabolic characteristics	Urinary protein	Renal function characteristics	Structural changes in the kidney	Alterations in renal circulation	References
Nicotinamide(NA)120mg/kg /Streptozotocin(STZ)60mg/kg	C57BL/6J mice	T2DM	Urine volume, food consumption, water intake, body weight, and HbA1c levels were significantly increased	8 fold increase after 5 weeks (vs. Normal Control)	Uric acid, serum urea, BUN, and creatinine levels were significantly increased, showing a 2- to 3-fold increase after 5 weeks compared to the normal control.	Perivascular lymphocytic aggregates, endotheliosis, inflammatory cell infiltration, degenerative changes, fibrotic, interstitial hemorrhage, and glomerular necrosis.	Unknown	(225)
High Fat Diet (HFD) mice	C57BL/6J mice	T2DM	1) The increase of body and kidney weights. 2) Impaired glucose tolerance, hyperinsulinemia, polyuria.	2.7-fold increase in 24-h urinary albumin excretion.	1) The increase of UACR after 8 weeks. 2) eGFR increase at 12 week and reduce at 16 weeks.	1) 20% increase in renal glomerular volume. 2) 18% increase in renal collagen deposition. 3) 8% drop of glomerular podocytes.	1) Suppress the velocity of Vs (renal arteries peak systolic velocity). 2) Decreases in Vd(renal arteries minimal end diastolic velocity) after 12 weeks.	(226, 227)
STZ + HFD mice	C57BL/6J mice	T2DM	1) The increase of body and kidney weights. 2) The increase of fasting blood glucose and cholesterol.	1) 2.37–fold increase in UACR.	1) 2.37–fold increase in UACR. 2) The magnitude of the urine cystatin C-to-creatinine ratio increased.	1) Glomerular vasodilation and mesangial expansion. 2) Angiogenesis is aberrantly activated in the kidneys.	Reduction in renal medullary blood flow after 6 weeks	(228, 229)
BTBR-ob/ob mice	BTBR + C57BL/6	T2DM	Insulin resistance, hyperglycemia, hyperphagia, and obesity were observed.	A 2-fold increase was observed at 8 weeks, and a 10-fold increase at 20 weeks compared to BTBR WT.	Unknown	Progressive renal damage, hypertrophy, and accumulation of mesangial matrix were observed at 8 weeks, followed by glomerular lesions at 20 weeks.	Unknown	(230, 231)
ob/ob mice (leptindeficient)	C57BL/6J mice	T2DM	Obesity, hyperglycaemia and insulin resistance	1) developed proteinuria beginning at 4 weeks. 2) A 4-fold increase was observed after 22 weeks compared to WT mice.	Unknown	Glomerular hypertrophy and mesangial expansion at 8 weeks.	An increase in mean PI (pulsatility index) values (1.50 ± 0.13 vs. 1.18 ± 0.19) and mean RI (resistive index) values (0.81 ± 0.04 vs. 0.69 ± 0.06) was observed compared to lean mice	(230, 232)
db/db mice (leptinreceptor deficient)	C57BLKs/J mice	T2DM	1) The increase of body weight, kidney weight, blood glucose, blood lipids and insulin at 10 weeks. (vs.db/m)	Significantly increase of urinary albumin excretion at 8 weeks.	A 2-fold increase in UACR was observed compared to db/m mice.	1) Promoted the expression of fibrosis associated protein-collagen I in kidneys. 2) Exacerbated ROS formation in kidneys. 3) Promoted mouse renal apoptosis.	Significantly larger baseline afferent arteriole (AA) diameters.	(233–235)
KKAy mice	KKAy mice	T2DM	1) Reduced activity, lackluster coat, drank more, consumed more food, and more urinary output. 2) The increase of body weight, kidney weight, blood glucose.	24-hour urine protein levels were significantly higher than in C57BL/6J mice at 14 weeks.	A significant increase in BUN and serum creatinine was observed compared to C57BL/6J mice.	1) Glomerular hypertrophy, mesangial expansion, glomerular epithelial cell proliferation, renal interstitial inflammatory cell infiltration, partial renal tubular atrophy. 2) Irregular thickening of the GBM, effacement of the foot processes, accumulation of the mesangial and renal interstitial matrix	1) An increase in diameters of the glomerulus, afferent arterioles, glomerular capillaries and efferent arterioles. 2) Increased blood flow into the glomerulus.	(236)
eNOS deficiency mice	C57BLKs/J mice	T2DM	Body weights, urinary output, fasting blood glucose significantly increase at 8 weeks.	1) Albuminuria starting at12 weeks.	1) The significantly increase of serum creatinine and BUN at 24 weeks.	Renal hypertrophy, mesangial expansion, glomerulosclerosis, glomerular and interstitial fibrosis.	1) renal blood flow (RBF) was significantly decreased. 2) Mean arterial pressure (MAP) was significantly higher. 3) renal vascular resistance (RVR) was much higher	(237, 238)
NONcNZO10/LtJ mice	NON/LtJ+NZO/H1Lt	T2DM	Insulin resistance, maturity-onset hyperglycemia, visceral obesity, dyslipidemia.	Albuminuria	Serum creatinine levels were increased at 6 months.	Glomerulosclerosis, intraglomerular capillary thrombi and lipid deposition, nephritis, and Ig deposition.	Unknown	(239)
STZ	Zebrafish	T2DM	Unknown	Unknown	Unknown	Thickening of the glomerular basement membrane.	Unknown	(240)
alloxan (ALX)+HFD	Rabbits	T2DM	Unknown	24-hour urinary protein significantly increased in the DN group at different time-points.	Serum creatinine and urea nitrogen levels of the DN group increased at the end of the 8th week.	1) Lipid deposition and increased. 2) Increased renal medulla perfusion.	Renal Blood Flow (RBF) Values was decreased but no statistically significant difference.	(241, 242)
Goto-Kakizaki (GK)	rats	T2DM	The increase of kidney weight and blood glucose.	Increased of proteinuria.	1) Serum creatinine levels were significantly elevated. 2) BUN levels were similarly increased 2 to 5-fold.	Glomerular hypertrophy, GBM thickening, segmental glomerulosclerosis, tubulointerstitial fibrosis.	Blood flow and velocity increased in the renal microvasculature of GK rats, leading to abnormal renal hemodynamics characterized by high perfusion and high filtration.	(243–245)
OLETF	rats	T2DM	The increase of body weight and blood glucose.	Proteinuria progressively increased after the 20th week.	Increased of GFR.	1) Glomerular hypertrophy and GBM thickening. 2) Extracellular matrix expansion. 3) Nodular lesions, diffuse glomerulosclerosis, tubulo-interstitial fibrosis.	Total renal blood flow (RBF), superficial blood flow (SBF), and deep renal cortical blood flow (DBF) showed stepwise reductions.	(246–248)
ZDF	rats	T2DM	The increase of body weight and Fasting blood glucose.	Heavy proteinuria.	Unknown	glomerulosclerosis, tubulo-interstitial and vascular damage.	1) Increased mean PI and mean RI values compared to Zucker lean rats. 2) Increased in average SBP and DBP.	(246, 249)
ZSF1	rats	T2DM	The increase of body weight and blood glucose.	Proteinuria showed a progressive increase after the 12th week.	Reduced of GFR.	Glomerulosclerosis, severe tubulo-interstitial damage, and vascular damage.	1) Significant increase in average BP. 2) Reduction in renal blood flow and GFR.	(246, 250)
High-fat diet in low dose-STZ-treated, Heminephrectomies	rats	T2DM	1) Insulin resistance. 2) TC and TG were significantly increased.	Overt proteinuria was seen after 20 weeks.	Ccr(Creatinine clearance rate) was significantly higher at 20 weeks, but thereafter started to decline.	1) Diffuse mesangial proliferation with focal glomerulolysis and scattered global sclerosis. 2) Mesangial matrix proliferation and interstitial edema.	Unknown	(251)
BB rat	rat	T1DM	The increase of kidney weight and blood glucose.	Urinary albumin excretion of diabetic rats did not exceed the levels found in control.	Unknown	Enhanced GFR and thickening of GBM.	Glomerular filtration rates and renal blood flow were 43% and 48% greater in BB rats than in control rats.	(252, 253)
Uni-nephrectomies rat model of STZ induced DN	rat	T1DM	Unknown	Albuminuria	Unknown	1) GBM thickening, Fibrosis. 2) Mononuclear inflammatory cell infiltration.	Unknown	(216)
Akita mice: (Ins2 +/ C96Y) on C57Bl/6	mice	TIDM	1) Sustained hyperglycemia and polyuria.	Urinary albumin excretion significantly increased by 8 weeks.	Urinary excretion of nephrin increased at 16 and 20 weeks.	1) The increase of kidney size after 12 weeks. 2) Mesangial matrix expansion and GBM thickening. 3) Podocyte apoptosis.	Systolic blood pressure (BP) was significantly elevated at 10 weeks.	(254)
NOD mice	mice	TIDM	The increase of kidney weight and blood glucose.	Significantly increased albuminuria.	Unknown	Enlarged glomeruli and mesangial sclerosis.	Systolic blood pressure (SBP) was significantly higher in NOD mice compared to control group mice.	(255)
HD-OVE	mice	TIDM	The increase of kidney weight and blood glucose.	1) Significant 3-fold increase in ACR versus WT.	HD-OVE mice exhibited hyperfiltration levels of GFR at 12 weeks of age, by 20 weeks, showed significant GFR reductions.	1) Glomerular hypertrophy and mesangial matrix expansion. 2) Renal tubulointerstitial fibrosis and elevated α-SMA. 3) Increased collagen and fibronectin production.	1) Systolic BP is progressively increased.	(256)
Alloxan	Dogs	TIDM	Polyuria and hyperglycemia were observed, and HbA1c and urine glucose levels were significantly higher than normal.	The prevalence of microalbuminuria of 59% in the present study.	1) Significantly increased of GFR and RPF. 2) BUN levels being fourfold greater than normal.	1) The area of glomerulus and area ratio between glomerulus and renal capsule were decreased. 2) The collagen fibers and glycogen in kidney cortex were significantly accumulation.	1) The highest recorded prevalence of systolic and diastolic hypertension was 55 and 64%. 2) Significantly increased of GFR and RPF.	(256–258)

### 4.1 T1DM

Type 1 diabetes mellitus (T1DM) is an autoimmune disorder characterized by pancreatic β-cell destruction and subsequent absolute insulin deficiency. Commonly employed animal models for T1DM research include spontaneous non-obese diabetic (NOD) mice, BioBreeding (BB) rats, streptozotocin (STZ)-induced models, and transgenic models. The following section briefly outlines their modeling mechanisms.

Streptozotocin (STZ) selectively destroys pancreatic β-cells through necrosis or apoptosis, thereby inducing absolute insulin deficiency, and is widely used for establishing T1DM in rodents. The pathological manifestations of STZ-induced diabetes typically include sustained hyperglycemia, albuminuria, thickening of the glomerular basement membrane, and mesangial expansion, but rarely present with hypertension or glomerulosclerosis (210). Similarly to streptozotocin, alloxan induces T1DM and associated complications in Beagle dogs through selective destruction of pancreatic β-cells (211).

The Akita mice model develops T1DM through an Ins2 gene point mutation (Cys96Tyr) that causes insulin protein misfolding, triggering endoplasmic reticulum stress and subsequent pancreatic β-cell apoptosis, ultimately resulting in absolute insulin deficiency. This model faithfully recapitulates the progressive β-cell failure characteristic of human T1DM (212). In contrast, non-obese diabetic (NOD) mice exhibit autoimmune destruction of pancreatic β-cells mediated by autoreactive T cells, driven by genetic susceptibility associated with the H-2g7 MHC haplotype. Aberrant presentation of islet antigens through defective MHC class II molecules activates both CD4+ and CD8+ T cells, mimicking the autoimmune pathogenesis observed in human T1DM (213).

The OVE26 mice model develops T1DM through pancreatic β-cell-specific overexpression of calmodulin, resulting in β-cell damage and absolute insulin deficiency. This model demonstrates characteristic DKD manifestations including mesangial matrix expansion, significant proteinuria, podocyte loss, glomerular hypertrophy, and tubulointerstitial fibrosis. Notably, female OVE26 mice exhibit more pronounced DKD manifestations compared to males. This model faithfully recapitulates multistage pathological features of human DKD while partially reflecting gender-specific disease progression patterns (214).

BB rats represent a well-established rodent model of T1DM, characterized by spontaneous autoimmune-mediated destruction of pancreatic β-cells leading to absolute insulin deficiency, thereby recapitulating key pathological features of human T1DM (215).

### 4.2 T2DM

The animal model of T2DM widely adopts multiple low-dose STZ injections to induce progressive pancreatic β-cell destruction, representing a well-established modeling approach that mimics β-cell dysfunction in human T2DM pathogenesis (216). In addition, experimental models of T2DM frequently employ genetically obese rodents, such as leptin-deficient (ob/ob) or leptin receptor-deficient (db/db) mice, to simulate metabolic dysregulation. These strains recapitulate key pathophysiological hallmarks observed in early human DKD, characterized by hyperglycemia, hyperinsulinemia, and progressive albuminuria (217). The HFD model induces insulin resistance through metabolic dysregulation characterized by excessive adipose accumulation and elevated free fatty acid (FFA) release, which collectively impair insulin signaling transduction. Concurrently, adipose tissue macrophage infiltration and pro-inflammatory cytokine secretion establish chronic low-grade inflammation, ultimately compromising pancreatic β-cell function and insulin secretion. This model effectively recapitulates two hallmark pathological features of human T2DM: systemic insulin resistance and progressive β-cell failure (218). The KK-Ay mouse model, carrying the dominant yellow obese Ay allele, develops hyperphagia, metabolic dysregulation, and obesity. Characteristic renal manifestations in this model include albuminuria, mesangial hyperplasia, segmental glomerulosclerosis, and podocyte depletion. Notably, KK-Ay mice maintain preserved renal function without progression to end-stage renal failure, establishing this model as particularly suitable for investigating early-to-mid-stage DKD pathogenesis (219). The NONcNZO10/LtJ murine model represents a polygenic T2DM characterized by development of insulin resistance with enhanced gluconeogenesis, progressing to moderate obesity and diabetes accompanied by visceral lipid deposition. This model show the intricate pathogenesis of human T2DM through polygenic interactions that mirror the multifactorial etiology of the disease (220). Rat models are increasingly employed in T2DM research, complementing mice systems. The Goto-Kakizaki (GK) rat model exhibits pancreatic β-cell dysfunction resulting from reduced GLUT2 expression and downregulation of SNARE complex components, coupled with mild insulin resistance, collectively (221). OLETF rats, characterized by a deficiency in the cholecystokinin-1 receptor (CCK-1R), exhibit hyperphagia and insulin resistance, progressing spontaneously to obese T2DM. The renal pathology in this model manifests through distinct morphological and functional alterations, including glomerular basement membrane thickening, mesangial matrix expansion, increased urinary albumin excretion, and tubular epithelial injury (222). ZSF1 rats, harboring inherited leptin signaling impairment, spontaneously develop obesity, hyperglycemia, hyperlipidemia, and mild hypertension, ultimately progressing to T2DM. This model demonstrates characteristic pathological manifestations including albuminuria, glomerular mesangial matrix expansion, and tubulointerstitial fibrosis (223). Zucker Diabetic Fatty (ZDF) rats, a substrain derived from obese Zucker rats, progress to T2DM. This diabetic model exhibits characteristic features including obesity, hyperglycemia, albuminuria, and glomerular hyperfiltration, accompanied by concurrent dilation of both afferent and efferent arterioles (224).

Additionally, zebrafish and rabbit models may be employed in modeling both type 1 and type 2 diabetic nephropathy. The pathological alterations and renal microcirculatory injury characteristics across these animal models are summarized in **Table 1**.

## 5 Techniques and methods for assessing renal microcirculation

The kidney contains a highly complex vascular system, and DKD is associated with glomerular vascular endothelial dysfunction and capillary injury. Therefore, methods and tools capable of monitoring vascular lesions and hemodynamic changes are needed to better understand the pathophysiological processes of DKD (**Table 2**).

**Table 2:** Techniques for assessing renal microcirculation function: advantages and limitations.

Methods	Applications	Advantages	Disadvantages	References
SR	1) Visualizing glomeruli, renal arterioles, proximal tubules. 2) Observing the diameter of renal vasculature while using contrast agent.	1) No geometric magnification of vessels. 2) Highly precise X-ray beam, small X-ray source and non-invasive method. 3) Micron-sized arteriole can be observed.	Difficulty in reflecting the internal three-dimensional structure of the kidney.	(260, 261)
BOLD-MRI	Measurement of deoxyhemoglobin levels to reflect the degree of tissue hypoxia, thus indirectly assessing renal microcirculation.	1) Noninvasive method. 2) Without exposure to radiation or exogenous contrast agents. 3) High spatial resolution.	1) Difficulties in manual tracing of appropriate ROIs. 2) Numerous analytical techniques, no standardized methodology.	(285)
μCT	1) Quantitative monitoring of renal microcirculation, changes in vascular structure and vascular function. 2) Renal vasculature from a 3D perspective.	1) Noninvasive method. 2) Quantitative monitoring. 3) Showing the 3D structure of the renal microcirculation.	1) X-ray exposure; 2) The need for iodine- based contrast agents.	(268)
Electron microscopy	Observation of the ultrastructure of the glomerular interior such as podocytes and endothelium.	1) Ultrastructural changes in the renal microcirculation can be observed.	1) Sacrifice of the animals.	(299)
fluorescence microangiography	Detect, quantitate and analyze the changes in renal microvasculature.	1) Define perfused capillaries and their precise architecture.	1) Sacrifice of the animals.	(275, 300)
MPM	Quantify multiple dynamic renal processes, including capillary flow, permeability, and glomerular function. 2) Study of intracellular changes and crosstalk between cells.	1) Noninvasive method. 2) High-resolution(cellular and subcellular resolutions).	1) Labor-intensive and time-consuming. 2) Affected by movement. 3) Effects of anesthesia.	(301–303)
MRI	1) Detect and analyze the changes in renal microvasculature. 2) Observation of ultrastructural changes in the kidney. 3) Analyze renal function.	1) Noninvasive method. 2) Effectively evaluate fibrosis in the kidney.	Affected by movement.	
IVIM-MRI	1) Valuation of renal microcirculation by pure molecular diffusion (D), pseudo-diffusion coefficient (D*), perfusion fraction (f), mean diffusion (MD), and mean kurtosis (MK). 2) Shows microstructural and functional changes in tissues.	1) Noninvasive method. 2) Without exposure to radiation or exogenous contrast agents.	1) Affected by movement. 2) Affected by no Gaussian diffusion behavior and Rician noise.	(281, 282)
MRE-MRI	1) Detecting the elasticity, stiffness, and structure of kidney tissue to determine the extent of renal fibrosis. 2) Indirect detection of kidney perfusion.	1) Noninvasive method. 2) Without exposure to radiation or exogenous contrast agents. 3) Can assess the extent of renal fibrosis.	1) Affected by movement. 2) Limitations in testing kidney function.	(291)
ASL-MRI	Measurement of renal blood perfusion.	1) Noninvasive method. 2) Without exposure to radiation or exogenous contrast agents. 3) Repeatable and stable.	1) Affected by movement. 2) low signal-to-noise ratio.	(304, 305)
CEUS	1) Quantitative assessment of renal microcirculation and microvascular perfusion characteristics. 2) Analyze renal function. 3) Assess renal transplant viability.	1) Radiation-free and non-nephrotoxic. 2) Distinguishes perfusion heterogeneity between renal cortex and medulla, enabling detection of microvascular abnormalities. 3) Real-time imaging capability.	1) Operator-dependent, requiring experienced personnel for optimal acquisition. 2) Outcomes may vary across ultrasound machine models, necessitating protocol standardization.	(298)

### 5.1 Synchrotron radiation

The main characteristics of SR are its high intensity and broad-band energy spectrum, which make it valuable in medical research (259). Additionally, SR offers higher resolution compared to conventional X-rays (259). These advantages enable it to be particularly useful for imaging vascular structures smaller than 100 μm in diameter and visualizing intricate structures within renal units (260). A study demonstrated that angiography of rat arteries using synchrotron radiation successfully observed four levels of branching in the renal arteries, with resting diameters ranging from 28 to 400 μm (261). The main limitation of this technique is its difficulty in accurately reflecting the three-dimensional structure inside the kidney (262).

### 5.2 Microcomputed tomography

The principle of μCT application is based on the attenuation of X-rays as they pass through the imaged object. This technique is now widely used for the quantitative evaluation of cardiac, bone, and soft tissue structures (263). μCT enables the visualization and quantitative analysis of blood vessels in three dimensions (264) and has been employed to study blood supply to tumors, vascular calcification, and vascular regeneration (265–267). A study demonstrated that μCT can provide stable, noninvasive monitoring of blood vessels in a mouse model, reflecting changes in renal blood volume, as well as renal vessel diameter, branching, and tortuosity (268). However, this method also has some limitations, such as the risk of exposure to X-rays and the need for iodine-based contrast agents.

### 5.3 Electron microscopy

Glomerular endothelial cell injury plays a crucial role in the progression of glomerular disease. In progressive kidney diseases such as DKD, angiogenesis is impaired due to endothelial cell injury, leading to sclerosis in the affected areas (269). Additionally, it has been demonstrated that mice models with site-specific renal microvascular endothelial injury are more prone to thrombosis in glomerular and peritubular microvessels, which subsequently affects renal microcirculation (270). Electron microscopy is an effective method for visualizing fine ultrastructure and detecting changes in endothelial cells. It can reveal the separation of endothelial cells from the glomerular basement membrane, the loss of glomerular endothelial cells, and glomerulosclerosis in chronic glomerular lesions (271). Additionally, electron microscopy can observe the loss of podocytes due to renal ischemia (272). These findings highlight the potential of electron microscopy to assess renal microcirculation. However, this method often requires the sacrifice of animals and may be subject to sampling errors (273).

### 5.4 Fluorescence microangiography

Fluorescence microangiography is a simple and widely applicable technique for assessing renal microcirculation and effectively delineating the renal microvasculature in three dimensions (274). Additionally, fluorescence microangiography allows for the quantitative assessment of renal microvascular changes. One study demonstrated a 40% ± 7.4% reduction in the number of peritubular capillaries, a 36% ± 4% reduction in individual capillary cross-sectional area, and a 62% ± 2.2% reduction in total peritubular perfusion 8 weeks after AKI. Thus, fluorescence microangiography has distinct advantages in detecting capillary perfusion and its ultrastructure (275).

### 5.5 Intravital multiphoton microscopy

Intravital multiphoton microscopy technology enables the tracking and detection of single cells in living organisms, making it particularly well-suited for studying cellular and molecular changes during the progression of chronic diseases (276). Additionally, this technology supports the simultaneous study of renal function and morphology. It allows for the visualization of renal structures and cells, including the glomerular and peritubular vascular systems, podocytes, mesangial cells, endothelial cells, and endothelial glycocalyxes (277). In the study of renal hemodynamics, intravital multiphoton microscopy detected renal capillary diameters of 8.7 ± 0.5 μm in 5/6 nephrectomy rats, which increased to 10.1 ± 1.3 μm after two weeks. This technique also enabled the quantification of the average cross-sectional blood velocity and the volume flow rate of the renal capillaries (278). However, altered renal hemodynamics induced by general anesthesia, animal movement, and nephron heterogeneity may affect the imaging results of intravital multiphoton microscopy (279).

### 5.6 MRI

#### 5.6.1 Intravoxel incoherent motion imaging-MRI

IVIM-MRI has its origins in diffusion MRI, where IVIM refers to translational movements within a given voxel that, during the measurement time, present a distribution of speeds in orientation and/or amplitude (280). IVIM-MRI can provide information on tissue microcirculation as well as blood flow, making it highly valuable for studying tumor blood supply and renal microvasculature (281). In practice, IVIM-MRI can quantitatively assess renal microcirculation in rats with diabetic nephropathy by evaluating pure molecular diffusion (D), pseudo-diffusion coefficient (D*), perfusion fraction (f), mean diffusion (MD), and mean kurtosis (MK) (282). However, the use of contrast media may affect renal structure and function, a consideration that should be the focus of future studies (282).

#### 5.6.2 Blood oxygen level-dependent-MRI

The basic principle of BOLD-MRI is that changes in renal tissue deoxyhemoglobin concentrations generate phase incoherence of magnetic spins, leading to an increase in the apparent relaxation rate R2* (283). Renal microcirculatory pathology is often associated with alterations in renal perfusion, which subsequently results in tissue hypoxia. Therefore, BOLD-MRI, as a noninvasive technique to assess renal oxygenation, can indirectly reflect the status of renal microcirculation (284). In specific studies, R2* serves as an estimate of tissue oxygenation, with lower R2* values indicating higher tissue oxygenation. Additionally, as the strength of the magnetic field increases, the change in R2* value is more pronounced, effectively improving the stability and sensitivity of BOLD-MRI (285). Although there are various analytical techniques for BOLD-MRI, a unified standard method has not yet been established, which may lead to inconsistencies in analytical results.

#### 5.6.3 Magnetic resonance elastography-MRI

Organ stiffness is altered in various diseases, such as cirrhosis and renal fibrosis. Therefore, the quantitative assessment of tissue stiffness can be valuable in studying disease progression. MRE-MRI, a technique that detects tissue stiffness within the body, was initially used for the evaluation of liver fibrosis. As this technology has advanced, it has become a viable alternative to liver biopsy for the diagnosis of cirrhosis (286). Today, the application of this technology is gradually expanding to include kidney research. A study demonstrated that the average stiffness of the renal parenchyma was 4.35 kPa in normal subjects and 5.10 kPa in CKD patients, regardless of disease stage (287). Additionally, measurements of kidney stiffness can serve as a predictive indicator of renal function decline (288). In assessing renal microcirculation, one study found that renal medulla stiffness was inversely related to renal blood flow (289). Another study found that renal cortical stiffness decreased as renal blood flow decreased, suggesting that the renal blood flow profile may mask the presence of renal fibrosis (290). This finding is consistent with another study, which showed that as chronic kidney disease worsens, kidney stiffness is paradoxically reduced (291). Due to the diversity of relationships between renal stiffness and renal blood flow, MRE-MRI can only be used as an indirect method to assess renal microcirculation.

#### 5.6.4 Arterial spin labeling-MRI

The principle of ASL-MRI involves using water protons in the blood as tracers. Water protons are magnetized and tagged before entering the target tissues, and signals are acquired when these labeled water protons pass through the arterial vasculature and reach the imaging plane, producing the labeled image. Simultaneously, control images without applied magnetic markers are acquired in the same imaging plane. The difference in signal intensity between the two images reflects tissue perfusion, allowing for an understanding of hemodynamic and microcirculatory changes during the disease process (292). This technique was initially used in cerebrovascular-related diseases and has shown great potential, leading to its widespread application in perfusion imaging for chronic kidney disease (292). A study demonstrated that MRI can quantitatively compare changes in renal cortical and medullary blood flow between patients with acute kidney injury and normal subjects (293). In mice, MRI can also detect renal blood flow. One study showed that blood flow to the kidney on the side with a clamped renal pedicle dropped to 412 ± 46 mL/min (moderate) and 239 ± 48 mL/min (severe) within 28 days compared to a normal kidney (294). Although this method does not require a contrast agent and offers high reproducibility, it has the disadvantages of a low signal-to-noise ratio and slow temporal and spatial resolution (292).

### 5.7 Contrast-enhanced ultrasound

CEUS is an advanced ultrasonographic technique that utilizes ultrasound contrast agents (UCAs) to achieve detailed visualization of anatomical and vascular structures, including the depiction of renal microcirculation. CEUS enables precise imaging through the unique physical properties of UCAs and their hemodynamic contrast mechanisms. Briefly, UCAs (e.g., SonoVue^®^) consist of inert gas encapsulated within a phospholipid/protein shell, with a diameter of 2.5–3 micrometers (μm) (295). This allows them to traverse capillaries and microvasculature unimpeded. Under low mechanical index (MI <0.1) ultrasound fields, microbubbles undergo nonlinear oscillations, emitting harmonic signals. These signals are captured via harmonic-specific imaging techniques, enabling dynamic assessment of tissue microcirculation (296). CEUS demonstrates superior spatial resolution compared to conventional ultrasound. Regarding safety, the inert gas component is eliminated via pulmonary exhalation, while the shell components undergo hepatic metabolism, ensuring non-nephrotoxicity and absence of tissue deposition, with an excellent safety profile (297). In clinical practice, the mechanism of CEUS enables its application in assessing microcirculation impairment in renal tissues. Its radiation-free nature, cost-effectiveness, and repeatability make it particularly advantageous for long-term monitoring of patients with renal insufficiency, positioning CEUS as a valuable complement or alternative to CT/MRI (298).

## 6 Application of new techniques in the study of microcirculation in DKD

In DKD, the renal microcirculation can undergo various lesions. Currently, methods and techniques for studying the mechanisms underlying renal microcirculation pathology are still evolving. The following section describes several common methods used to investigate the mechanisms of renal pathology.

### 6.1 Single-cell RNA-sequencing

Single-cell sequencing (scRNA-seq) has emerged as a state-of-the-art method for revealing the heterogeneity and complexity of RNA transcription within individual cells, as well as for identifying different cell types and functions within tissues. This technique is therefore valuable for exploring the expression of specific markers and genes across a wide range of cells within the kidney. Given the critical role of GECs in the renal microcirculatory system, scRNA-seq is widely recognized for its ability to explore endothelial cell heterogeneity. It has been demonstrated that under pathological conditions, such as tumorigenesis, quiescent endothelial cells are activated and become involved in neovascularization and disease progression (306). In the kidney, scRNA-seq has been used to analyze more than 40,000 mouse renal endothelial cells, revealing the extensive heterogeneity of these cells across the cortex, glomerulus, and medulla, as well as changes in gene expression in response to hypertonicity or dehydration (307). Additionally, scRNA-seq can detect changes in the distribution and number of cells within the glomerulus. It has been demonstrated that the proportion of GECs in the glomeruli of diabetic mice is significantly higher, while the proportion of mesangial cells (MCs) and podocytes is reduced compared to normal mice (308). From a genetic perspective, single-cell sequencing technology can be used to explore and screen key genes associated with DKD. Through GSEA analysis and other approaches, researchers can study the specific signaling pathways of these key genes and the mechanisms by which they influence DKD (309). In one study, researchers found that MRTF-SRF transcriptional regulation was activated in mesangial cells (MCs) in DKD, affecting the expression of the downstream VEGFA-VEGFR2 signaling pathway and the PDGFRB pathway, which may contribute to glomerular hyperfiltration in DKD. This finding was further supported by *in vitro* experiments, demonstrating the usefulness of scRNA-seq technology in guiding future research (310). In summary, scRNA-seq plays a crucial role in studying the mechanisms of renal microcirculatory lesions in DKD by revealing the gene structure and expression status of individual cells and reflecting intercellular heterogeneity through high-throughput sequencing analyses of genomes, transcriptomes, and epigenomes at the single-cell level. However, scRNA-seq also has several limitations: 1) Isolating glomerular cells is challenging; 2) No effective standardized pipelines are currently available; 3) It does not comprehensively show all cell markers; 4) There is no harmonized methodology for analysis and statistics, requiring variation based on the choice of calculation tools and databases (66, 309); 5) It is difficult to explore information about the spatial location of gene expression (311).

### 6.2 Spatial transcriptomics

To fully understand the gene function of cells within the kidney and the signaling pathways that facilitate crosstalk between cells, it is essential to explore the extent, timing, and spatial location of relevant gene expression. However, scRNA-seq disrupts cellular structural organization and fails to provide spatial information. The emergence and development of ST have enabled the dual determination of both quantification and localization of target genes (311). ST technologies broadly fall into two categories: imaging-based and sequencing-based. Imaging-based ST can provide single-cell or subcellular resolution with high RNA capture efficiency, while sequencing-based ST technologies offer whole transcriptome analysis, albeit with relatively lower RNA capture efficiency (311). In practice, ST has been performed on renal biopsy specimens from DKD patients to obtain gene expression profiles of renal tissues. Gene expression in the glomerular neovascularization region revealed increased expression of genes involved in angiogenic signaling, endothelial cell proliferation, and neointimal maturation, elucidating potential mechanisms of glomerular neovascularization in DKD (312). The advantages of spatial transcriptomics are clear, but several caveats remain: 1) Requirement for high RNA integrity; 2) Resolution and RNA capture rates vary across different spatial platforms; 3) Imaging-based techniques analyze a limited number of genes (311).

## 7 Conclusion

**Figure 3:**
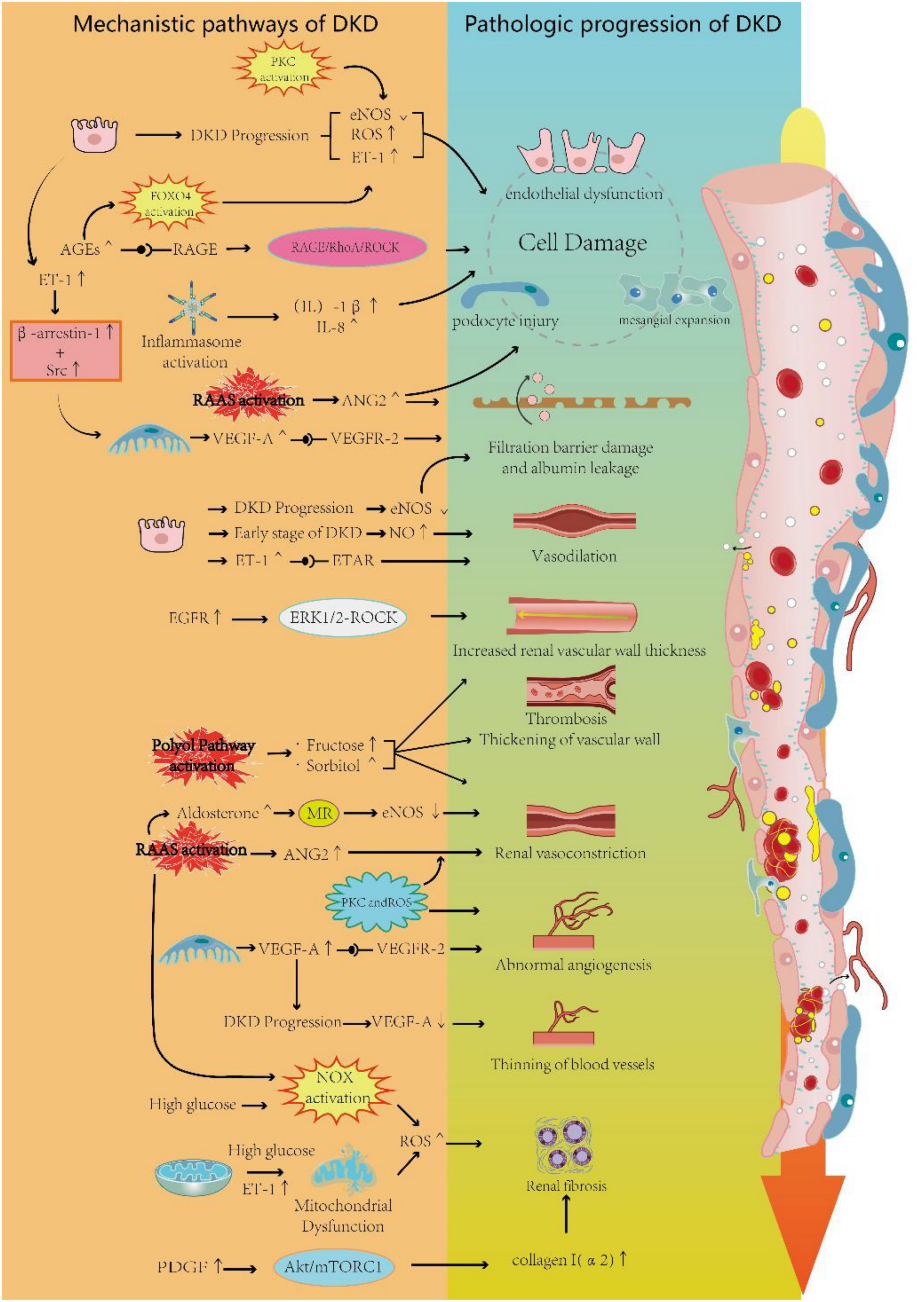
Renal microcirculation pathological changes of DKD. This schematic delineates the pathological mechanisms underlying microcirculatory dysfunction in DKD. The figure is organized into two interconnected sections: Mechanistic Pathways of DKD and Pathologic Progression of DKD. The Mechanistic Pathways section illustrates molecular drivers of microvascular injury, including signaling cascades and cellular interactions. The Pathologic Progression section highlights structural and functional alterations observed in advanced DKD. Cross-referenced pathways between these sections elucidate the dynamic interplay between molecular events and clinical manifestations, providing a comprehensive overview of DKD pathogenesis.

In conclusion, this review summarizes the lesions in the renal microcirculatory system in DKD and the mechanisms involved (**Figure 3**), with a focus on the alterations in the structure of renal microvessels and the dysfunction of renal vascular endothelial cells. The evidence we have presented sheds light, to some extent, on the mechanisms underlying renal microcirculatory pathology in DKD, highlighting the potential for developing therapeutic strategies by targeting these pathways and mechanisms. However, these mechanisms are relatively complex and do not function independently; rather, they interact to form a complex network of signaling pathways. Therefore, it is essential to continue elucidating these intricate processes and interrelationships, necessitating further exploration in this field. Additionally, we have summarized the animal models, assessment methods, and detection techniques that may be used to study renal microcirculatory lesions in DKD, with the aim of providing a reference for DKD-related research.

## References

[1] Sun H Saeedi P Karuranga S Pinkepank M Ogurtsova K Duncan BB. IDF Diabetes Atlas: Global, regional and country-level diabetes prevalence estimates for 2021 and projections for 2045. Diabetes Res Clin Pract. (2022) 183:109119. doi: 10.1016/j.diabres.2021.109119 34879977 PMC11057359

[2] Alicic RZ Rooney MT Tuttle KR. Diabetic kidney disease: challenges, progress, and possibilities. Clin J Am Soc Nephrol. (2017) 12:2032–45. doi: 10.2215/cjn.11491116 PMC5718284 28522654

[3] Vučić Lovrenčić M Božičević S Smirčić Duvnjak L. Diagnostic challenges of diabetic kidney disease. Biochem Med (Zagreb). (2023) 33:30501. doi: 10.11613/bm.2023.030501 PMC10373061 37545693

[4] Tan KS McDonald S Hoy W. The diagnostic performance of a clinical diagnosis of diabetic kidney disease. Life (Basel). (2023) 13:1492. doi: 10.3390/life13071492 37511866 PMC10381424

[5] Wu Y Xiong T Tan X Chen L. Frailty and risk of microvascular complications in patients with type 2 diabetes: a population-based cohort study. BMC Med. (2022) 20:473. doi: 10.1186/s12916-022-02675-9 36482467 PMC9733051

[6] Tateyama Y Shimamoto T Uematsu MK Taniguchi S Nishioka N Yamamoto K. Status of screening and preventive efforts against diabetic kidney disease between 2013 and 2018: analysis using an administrative database from Kyoto-city, Japan. Front Endocrinol (Lausanne). (2023) 14:1195167. doi: 10.3389/fendo.2023.1195167 37576956 PMC10413551

[7] White S Chadban S. Diabetic kidney disease in Australia: current burden and future projections. Nephrol (Carlton). (2014) 19:450–8. doi: 10.1111/nep.12281 24888506

[8] Thomé GG Bianchini T Bringhenti RN Schaefer PG Barros EJG Veronese FV. The spectrum of biopsy-proven glomerular diseases in a tertiary Hospital in Southern Brazil. BMC Nephrol. (2021) 22:414. doi: 10.1186/s12882-021-02603-8 34903188 PMC8667371

[9] Shestakova MV Vikulova OK Zheleznyakova AV Isakov MA Dedov II. Diabetes epidemiology in Russia: what has changed over the decade]? Ter Arkh. (2019) 91:4–13. doi: 10.26442/00403660.2019.10.000364 32598625

[10] Detournay B Simon D Guillausseau PJ Joly D Verges B Attali C. Chronic kidney disease in type 2 diabetes patients in France: prevalence, influence of glycaemic control and implications for the pharmacological management of diabetes. Diabetes Metab. (2012) 38:102–12. doi: 10.1016/j.diabet.2011.11.005 22252014

[11] Galiyeva D Gusmanov A Sakko Y Issanov A Atageldiyeva K Kadyrzhanuly K. Epidemiology of type 1 and type 2 diabetes mellitus in Kazakhstan: data from unified National Electronic Health System 2014-2019. BMC Endocr Disord. (2022) 22:275. doi: 10.1186/s12902-022-01200-6 36368961 PMC9650815

[12] Cunningham A Benediktsson H Muruve DA Hildebrand AM Ravani P. Trends in biopsy-based diagnosis of kidney disease: A population study. Can J Kidney Health Dis. (2018) 5:2054358118799690. doi: 10.1177/2054358118799690 30263130 PMC6149029

[13] Hallan SI Øvrehus MA Romundstad S Rifkin D Langhammer A Stevens PE. Long-term trends in the prevalence of chronic kidney disease and the influence of cardiovascular risk factors in Norway. Kidney Int. (2016) 90:665–73. doi: 10.1016/j.kint.2016.04.012 27344204

[14] Heller T Blum M Spraul M Wolf G Müller UA. Diabetic co-morbidities: prevalences in Germany. Dtsch Med Wochenschr. (2014) 139:786–91. doi: 10.1055/s-0034-1369889 24691694

[15] Martínez-Castelao A De Alvaro F Górriz JL. Epidemiology of diabetic nephropathy in Spain. Kidney Int Suppl. (2005) 99:S20–24. doi: 10.1111/j.1523-1755.2005.09905.x 16336572

[16] Jayakumari S Gomez R Dipin S Jayakumar RV Vijayakumar K Sreenath R. Prevalence of normoalbuminuric chronic kidney disease among individuals with type 2 diabetes mellitus from India. Indian J Med Res. (2022) 156:632–9. doi: 10.4103/ijmr.IJMR_2595_19 PMC10231751 36926780

[17] Mihardja L Delima D Massie RGA Karyana M Nugroho P Yunir E. Prevalence of kidney dysfunction in diabetes mellitus and associated risk factors among productive age Indonesian. J Diabetes Metab Disord. (2018) 17:53–61. 10.1007/s40200-018-0338-6 PMC6013541 29984211

[18] Forbes JM Cooper ME. Mechanisms of diabetic complications. Physiol Rev. (2013) 93:137–88. doi: 10.1152/physrev.00045.2011 23303908

[19] Miranda M Balarini M Caixeta D Bouskela E. Microcirculatory dysfunction in sepsis: pathophysiology, clinical monitoring, and potential therapies. Am J Physiol Heart Circ Physiol. (2016) 311:H24–35. doi: 10.1152/ajpheart.00034.2016 27106039

[20] De Boer MP Meijer RI Wijnstok NJ Jonk AM Houben AJ Stehouwer CD. Microvascular dysfunction: a potential mechanism in the pathogenesis of obesity-associated insulin resistance and hypertension. Microcirculation. (2012) 19:5–18. doi: 10.1111/j.1549-8719.2011.00130.x 21883642

[21] Barrett EJ Liu Z. The endothelial cell: an “early responder” in the development of insulin resistance. Rev Endocr Metab Disord. (2013) 14:21–7. doi: 10.1007/s11154-012-9232-6 PMC3594570 23306779

[22] Chade AR. Small vessels, big role: renal microcirculation and progression of renal injury. Hypertension. (2017) 69:551–63. doi: 10.1161/hypertensionaha.116.08319 PMC5344725 28193706

[23] Guven G Hilty MP Ince C. Microcirculation: physiology, pathophysiology, and clinical application. Blood Purif. (2020) 49:143–50. doi: 10.1159/000503775 PMC7114900 31851980

[24] Horton WB Barrett EJ. Microvascular dysfunction in diabetes mellitus and cardiometabolic disease. Endocr Rev. (2021) 42:29–55. doi: 10.1210/endrev/bnaa025 33125468 PMC7846151

[25] Weil EJ Lemley KV Mason CC Yee B Jones LI Blouch K. Podocyte detachment and reduced glomerular capillary endothelial fenestration promote kidney disease in type 2 diabetic nephropathy. Kidney Int. (2012) 82:1010–7. doi: 10.1038/ki.2012.234 PMC3472108 22718189

[26] Bohle A Mackensen-Haen S Wehrmann M. Significance of postglomerular capillaries in the pathogenesis of chronic renal failure. Kidney Blood Press Res. (1996) 19:191–5. doi: 10.1159/000174072 8887259

[27] Schiffrin EL. Reactivity of small blood vessels in hypertension: relation with structural changes. State Art Lect Hyperten. (1992) 19:Ii1–9. doi: 10.1161/01.hyp.19.2_suppl.ii1-a 1735561

[28] Vallon V. Tubuloglomerular feedback and the control of glomerular filtration rate. News Physiol Sci. (2003) 18:169–74. doi: 10.1152/nips.01442.2003 12869618

[29] Artunc F Schleicher E Weigert C Fritsche A Stefan N Häring HU. The impact of insulin resistance on the kidney and vasculature. Nat Rev Nephrol. (2016) 12:721–37. doi: 10.1038/nrneph.2016.145 27748389

[30] Fan L Gao W Nguyen BV Jefferson JR Liu Y Fan F. Impaired renal hemodynamics and glomerular hyperfiltration contribute to hypertension-induced renal injury. Am J Physiol Renal Physiol. (2020) 319:F624–f635. doi: 10.1152/ajprenal.00239.2020 32830539 PMC7642882

[31] Noris M Remuzzi G. New insights into circulating cell-endothelium interactions and their significance for glomerular pathophysiology. Am J Kidney Dis. (1995) 26:541–8. doi: 10.1016/0272-6386(95)90505-7 7645567

[32] Palomo M Moreno-Castaño AB Salas MQ Escribano-Serrat S Rovira M Guillen-Olmos E. Endothelial activation and damage as a common pathological substrate in different pathologies and cell therapy complications. Front Med (Lausanne). (2023) 10:1285898. doi: 10.3389/fmed.2023.1285898 38034541 PMC10682735

[33] Jourde-Chiche N Fakhouri F Dou L Bellien J Burtey S Frimat M. Endothelium structure and function in kidney health and disease. Nat Rev Nephrol. (2019) 15:87–108. doi: 10.1038/s41581-018-0098-z 30607032

[34] Tousoulis D Simopoulou C Papageorgiou N Oikonomou E Hatzis G Siasos G. Endothelial dysfunction in conduit arteries and in microcirculation. Novel therapeutic approaches. Pharmacol Ther. (2014) 144:253–67. doi: 10.1016/j.pharmthera.2014.06.003 24928320

[35] Carracedo M Ericson E Ågren R Forslöw A Madeyski-Bengtson K Svensson A. APOL1 promotes endothelial cell activation beyond the glomerulus. iScience. (2023) 26:106830. doi: 10.1016/j.isci.2023.106830 37250770 PMC10209455

[36] Stinghen AE Gonçalves SM Martines EG Nakao LS Riella MC Aita CA. Increased plasma and endothelial cell expression of chemokines and adhesion molecules in chronic kidney disease. Nephron Clin Pract. (2009) 111:c117–126. doi: 10.1159/000191205 19147993

[37] Wang Z Zhang Z Li Y Zhang Y Wei M Li H. Endothelial-derived complement factor D contributes to endothelial dysfunction in Malignant nephrosclerosis via local complement activation. Hypertens Res. (2023) 46:1759–70. doi: 10.1038/s41440-023-01300-3 PMC10184087 37188751

[38] Gyarmati G Shroff UN Izuhara A Deepak S Komers R Bedard PW. Sparsentan improves glomerular hemodynamics, cell functions, and tissue repair in a mouse model of FSGS. JCI Insight. (2024) 9:e177775. doi: 10.1172/jci.insight.177775 39226116 PMC11466195

[39] Mauer M Caramori ML Fioretto P Najafian B. Glomerular structural-functional relationship models of diabetic nephropathy are robust in type 1 diabetic patients. Nephrol Dial Transplant. (2015) 30:918–23. doi: 10.1093/ndt/gfu279 PMC4438739 25183630

[40] Dalla Vestra M Saller A Bortoloso E Mauer M Fioretto P. Structural involvement in type 1 and type 2 diabetic nephropathy. Diabetes Metab. (2000) 26:4, 8–14. 10922968

[41] Dejana E Hirschi KK Simons M. The molecular basis of endothelial cell plasticity. Nat Commun. (2017) 8:14361. doi: 10.1038/ncomms14361 28181491 PMC5309780

[42] Basile DP Friedrich JL Spahic J Knipe N Mang H Leonard EC. Impaired endothelial proliferation and mesenchymal transition contribute to vascular rarefaction following acute kidney injury. Am J Physiol Renal Physiol. (2011) 300:F721–733. doi: 10.1152/ajprenal.00546.2010 PMC3064142 21123492

[43] Zeisberg EM Potenta SE Sugimoto H Zeisberg M Kalluri R. Fibroblasts in kidney fibrosis emerge via endothelial-to-mesenchymal transition. J Am Soc Nephrol. (2008) 19:2282–7. doi: 10.1681/asn.2008050513 PMC2588112 18987304

[44] Locatelli M Zoja C Conti S Cerullo D Corna D Rottoli D. Empagliflozin protects glomerular endothelial cell architecture in experimental diabetes through the VEGF-A/caveolin-1/PV-1 signaling pathway. J Pathol. (2022) 256:468–79. doi: 10.1002/path.5862 35000230

[45] Obeidat M Obeidat M Ballermann BJ. Glomerular endothelium: a porous sieve and formidable barrier. Exp Cell Res. (2012) 318:964–72. doi: 10.1016/j.yexcr.2012.02.032 22465480

[46] Salmon AH Satchell SC. Endothelial glycocalyx dysfunction in disease: albuminuria and increased microvascular permeability. J Pathol. (2012) 226:562–74. doi: 10.1002/path.3964 22102407

[47] Korakas E Ikonomidis I Markakis K Raptis A Dimitriadis G Lambadiari V. The endothelial glycocalyx as a key mediator of albumin handling and the development of diabetic nephropathy. Curr Vasc Pharmacol. (2020) 18:619–31. doi: 10.2174/1570161118666191224120242 31889495

[48] Yu H Song YY Li XH. Early diabetic kidney disease: Focus on the glycocalyx. World J Diabetes. (2023) 14:460–80. doi: 10.4239/wjd.v14.i5.460 PMC10236994 37273258

[49] Khramova A Boi R Fridén V Granqvist AB Nilsson U Tenstad O. Proteoglycans contribute to the functional integrity of the glomerular endothelial cell surface layer and are regulated in diabetic kidney disease. Sci Rep. (2021) 11:8487. doi: 10.1038/s41598-021-87753-3 33875683 PMC8055884

[50] Zhao XP Chang SY Liao MC Lo CS Chenier I Luo H. Hedgehog interacting protein promotes fibrosis and apoptosis in glomerular endothelial cells in murine diabetes. Sci Rep. (2018) 8:5958. doi: 10.1038/s41598-018-24220-6 29654303 PMC5899163

[51] Reidy K Kang HM Hostetter T Susztak K. Molecular mechanisms of diabetic kidney disease. J Clin Invest. (2014) 124:2333–40. doi: 10.1172/jci72271 PMC4089448 24892707

[52] Garg P. A review of podocyte biology. Am J Nephrol. (2018) 47:3–13. doi: 10.1159/000481633 29852492

[53] Kopp JB Anders HJ Susztak K Podestà MA Remuzzi G Hildebrandt F. Podocytopathies. Nat Rev Dis Primers. (2020) 6:68. doi: 10.1038/s41572-020-0196-7 32792490 PMC8162925

[54] Maezawa Y Takemoto M Yokote K. Cell biology of diabetic nephropathy: Roles of endothelial cells, tubulointerstitial cells and podocytes. J Diabetes Invest. (2015) 6:3–15. doi: 10.1111/jdi.12255 PMC4296695 25621126

[55] Wolf G Chen S Ziyadeh FN. From the periphery of the glomerular capillary wall toward the center of disease: podocyte injury comes of age in diabetic nephropathy. Diabetes. (2005) 54:1626–34. doi: 10.2337/diabetes.54.6.1626 15919782

[56] Avraham S Korin B Chung JJ Oxburgh L Shaw AS. The Mesangial cell - the glomerular stromal cell. Nat Rev Nephrol. (2021) 17:855–64. doi: 10.1038/s41581-021-00474-8 34508249

[57] Stockand JD Sansom SC. Regulation of filtration rate by glomerular mesangial cells in health and diabetic renal disease. Am J Kidney Dis. (1997) 29:971–81. doi: 10.1016/s0272-6386(97)90476-5 9186087

[58] Lakhe-Reddy S Li V Arnold TD Khan S Schelling JR. Mesangial cell αvβ8-integrin regulates glomerular capillary integrity and repair. Am J Physiol Renal Physiol. (2014) 306:F1400–1409. doi: 10.1152/ajprenal.00624.2013 PMC4059974 24740792

[59] Meek RL LeBoeuf RC Saha SA Alpers CE Hudkins KL Cooney SK. Glomerular cell death and inflammation with high-protein diet and diabetes. Nephrol Dial Transplant. (2013) 28:1711–20. doi: 10.1093/ndt/gfs579 PMC3707525 23314315

[60] Kriz W Löwen J Federico G van den Born J Gröne E Gröne HJ. Accumulation of worn-out GBM material substantially contributes to mesangial matrix expansion in diabetic nephropathy. Am J Physiol Renal Physiol. (2017) 312:F1101–f1111. doi: 10.1152/ajprenal.00020.2017 28228399

[61] Wang H Zhang R Wu X Chen Y Ji W Wang J. The wnt signaling pathway in diabetic nephropathy. Front Cell Dev Biol. (2021) 9:701547. doi: 10.3389/fcell.2021.701547 35059392 PMC8763969

[62] Marshall CB. Rethinking glomerular basement membrane thickening in diabetic nephropathy: adaptive or pathogenic? Am J Physiol Renal Physiol. (2016) 311:F831–f843. doi: 10.1152/ajprenal.00313.2016 27582102 PMC6121820

[63] Harindhanavudhi T Parks A Mauer M Caramori ML. Podocyte structural parameters do not predict progression to diabetic nephropathy in normoalbuminuric type 1 diabetic patients. Am J Nephrol. (2015) 41:277–83. doi: 10.1159/000381992 PMC4514533 26021523

[64] Ponchiardi C Mauer M Najafian B. Temporal profile of diabetic nephropathy pathologic changes. Curr Diabetes Rep. (2013) 13:592–9. doi: 10.1007/s11892-013-0395-7 23712652

[65] Löwen J Gröne EF Groß-Weißmann ML Bestvater F Gröne HJ Kriz W. Pathomorphological sequence of nephron loss in diabetic nephropathy. Am J Physiol Renal Physiol. (2021) 321:F600–f616. doi: 10.1152/ajprenal.00669.2020 34541901

[66] Hu S Hang X Wei Y Wang H Zhang L Zhao L. Crosstalk among podocytes, glomerular endothelial cells and mesangial cells in diabetic kidney disease: an updated review. Cell Commun Signal. (2024) 22:136. doi: 10.1186/s12964-024-01502-3 38374141 PMC10875896

[67] Cyr AR Huckaby LV Shiva SS Zuckerbraun BS. Nitric oxide and endothelial dysfunction. Crit Care Clin. (2020) 36:307–21. doi: 10.1016/j.ccc.2019.12.009 PMC9015729 32172815

[68] Baelde HJ Eikmans M Lappin DW Doran PP Hohenadel D Brinkkoetter PT. Reduction of VEGF-A and CTGF expression in diabetic nephropathy is associated with podocyte loss. Kidney Int. (2007) 71:637–45. doi: 10.1038/sj.ki.5002101 17264876

[69] Król-Kulikowska M Banasik M Kepinska M. The effect of selected nitric oxide synthase polymorphisms on the risk of developing diabetic nephropathy. Antioxidants (Basel). (2024) 13:838. doi: 10.3390/antiox13070838 39061907 PMC11273648

[70] Sivaskandarajah GA Jeansson M Maezawa Y Eremina V Baelde HJ Quaggin SE. Vegfa protects the glomerular microvasculature in diabetes. Diabetes. (2012) 61:2958–66. doi: 10.2337/db11-1655 PMC3478549 23093658

[71] Wang Y Nagase S Koyama A. Stimulatory effect of IGF-I and VEGF on eNOS message, protein expression, eNOS phosphorylation and nitric oxide production in rat glomeruli, and the involvement of PI3-K signaling pathway. Nitric Oxide. (2004) 10:25–35. doi: 10.1016/j.niox.2004.02.001 15050532

[72] De Vriese AS Stoenoiu MS Elger M Devuyst O Vanholder R Kriz W. Diabetes-induced microvascular dysfunction in the hydronephrotic kidney: role of nitric oxide. Kidney Int. (2001) 60:202–10. doi: 10.1046/j.1523-1755.2001.00787.x 11422752

[73] Shin Shin Y Baek SH Chang KY Park CW Yang CW Jin DC Kim YS. Relations between eNOS Glu298Asp polymorphism and progression of diabetic nephropathy. Diabetes Res Clin Pract. (2004) 65:257–65. doi: 10.1016/j.diabres.2004.01.010 15331206

[74] Kaneto H Katakami N Kawamori D Miyatsuka T Sakamoto K Matsuoka TA. Involvement of oxidative stress in the pathogenesis of diabetes. Antioxid Redox Signal. (2007) 9:355–66. doi: 10.1089/ars.2006.1465 17184181

[75] Palicz A Foubert TR Jesaitis AJ Marodi L McPhail LC. Phosphatidic acid and diacylglycerol directly activate NADPH oxidase by interacting with enzyme components. J Biol Chem. (2001) 276:3090–7. doi: 10.1074/jbc.M007759200 11060300

[76] Zhao HJ Wang S Cheng H Zhang MZ Takahashi T Fogo AB. Endothelial nitric oxide synthase deficiency produces accelerated nephropathy in diabetic mice. J Am Soc Nephrol. (2006) 17:2664–9. doi: 10.1681/asn.2006070798 PMC4618687 16971655

[77] Alhayaza R Haque E Karbasiafshar C Sellke FW Abid MR. The relationship between reactive oxygen species and endothelial cell metabolism. Front Chem. (2020) 8:592688. doi: 10.3389/fchem.2020.592688 33330380 PMC7732658

[78] Goycheva P Petkova-Parlapanska K Georgieva E Karamalakova Y Nikolova G. Biomarkers of oxidative stress in diabetes mellitus with diabetic nephropathy complications. Int J Mol Sci. (2023) 24:13541. doi: 10.3390/ijms241713541 37686346 PMC10488183

[79] Vodošek Hojs N Bevc S Ekart R Hojs R. Oxidative stress markers in chronic kidney disease with emphasis on diabetic nephropathy. Antioxidants (Basel). (2020) 9:925. doi: 10.3390/antiox9100925 32992565 PMC7600946

[80] Rajlic S Treede H Münzel T Daiber A Duerr GD. Early detection is the best prevention-characterization of oxidative stress in diabetes mellitus and its consequences on the cardiovascular system. Cells. (2023) 12:583. doi: 10.3390/cells12040583 36831253 PMC9954643

[81] Ren X Ren L Wei Q Shao H Chen L Liu N. Advanced glycation end-products decreases expression of endothelial nitric oxide synthase through oxidative stress in human coronary artery endothelial cells. Cardiovasc Diabetol. (2017) 16:52. doi: 10.1186/s12933-017-0531-9 28427390 PMC5397770

[82] Daehn IS. Glomerular endothelial cell stress and cross-talk with podocytes in early [corrected] diabetic kidney disease. Front Med (Lausanne). (2018) 5:76. doi: 10.3389/fmed.2018.00076 29629372 PMC5876248

[83] Mohan S Reddick RL Musi N Horn DA Yan B Prihoda TJ. Diabetic eNOS knockout mice develop distinct macro- and microvascular complications. Lab Invest. (2008) 88:515–28. doi: 10.1038/labinvest.2008.23 18391994

[84] Takahashi T Harris RC. Role of endothelial nitric oxide synthase in diabetic nephropathy: lessons from diabetic eNOS knockout mice. J Diabetes Res. (2014) 2014:590541. doi: 10.1155/2014/590541 25371905 PMC4211249

[85] Barton M Yanagisawa M. Endothelin: 30 years from discovery to therapy. Hypertension. (2019) 74:1232–65. doi: 10.1161/hypertensionaha.119.12105 31679425

[86] Schiffrin EL Pollock DM. Endothelin system in hypertension and chronic kidney disease. Hypertension. (2024) 81:691–701. doi: 10.1161/hypertensionaha.123.21716 38059359 PMC10954415

[87] Zhou Q Liu K Wu H Chen L Pouranan V Yuan M. Spironolactone rescues Dot1a-Af9-mediated repression of endothelin-1 and improves kidney injury in streptozotocin-induced diabetic rats. PloS One. (2012) 7:e47360. doi: 10.1371/journal.pone.0047360 23077601 PMC3471839

[88] Zanatta CM. Endothelin-1 levels and albuminuria in patients with type 2 diabetes mellitus. Diabetes Res Clin Pract. (2008) 80:299–304. doi: 10.1016/j.diabres.2007.12.024 18346810

[89] Li L. Targeting tissue-resident memory CD8(+) T cells in the kidney is a potential therapeutic strategy to ameliorate podocyte injury and glomerulosclerosis. Mol Ther. (2022) 30:2746–59. doi: 10.1016/j.ymthe.2022.04.024 PMC9372318 35514086

[90] Lenoir O. Direct action of endothelin-1 on podocytes promotes diabetic glomerulosclerosis. J Am Soc Nephrol. (2014) 25:1050–62. doi: 10.1681/asn.2013020195 PMC4005294 24722437

[91] Garsen M Lenoir O Rops AL Dijkman HB Willemsen B van Kuppevelt TH. Endothelin-1 induces proteinuria by heparanase-mediated disruption of the glomerular glycocalyx. J Am Soc Nephrol. (2016) 27:3545–51. doi: 10.1681/asn.2015091070 PMC5118481 27026367

[92] Qi H Casalena G Shi S Yu L Ebefors K Sun Y. Glomerular endothelial mitochondrial dysfunction is essential and characteristic of diabetic kidney disease susceptibility. Diabetes. (2017) 66:763–78. doi: 10.2337/db16-0695 PMC5319717 27899487

[93] Zou HH Wang L Zheng XX Xu GS Shen Y. Endothelial cells secreted endothelin-1 augments diabetic nephropathy via inducing extracellular matrix accumulation of mesangial cells in ETBR(-/-) mice. Aging (Albany NY). (2019) 11:1804–20. doi: 10.18632/aging.101875 PMC6461170 30926764

[94] Oshima M Shimizu M Yamanouchi M Toyama T Hara A Furuichi K. Trajectories of kidney function in diabetes: a clinicopathological update. Nat Rev Nephrol. (2021) 17:740–50. doi: 10.1038/s41581-021-00462-y 34363037

[95] Inscho EW Imig JD Cook AK Pollock DM. ETA and ETB receptors differentially modulate afferent and efferent arteriolar responses to endothelin. Br J Pharmacol. (2005) 146:1019–26. doi: 10.1038/sj.bjp.0706412 PMC1751231 16231007

[96] Žeravica R Čabarkapa V Ilinčić B Sakač V Mijović R Nikolić S. Plasma endothelin-1 level, measured glomerular filtration rate and effective renal plasma flow in diabetic nephropathy. Ren Fail. (2015) 37:681–6. doi: 10.3109/0886022x.2015.1010990 25687384

[97] Hofman C Francis B Rosenthal T Winaver J Rubinstein I Abassi Z. Effects of endothelin-1 on systemic and renal hemodynamics in hypertensive-diabetic rats (CRDH), diabetic rats (CDR), and hypertensive rats (SHR). J Cardiovasc Pharmacol. (2004) 44:S191–194. doi: 10.1097/01.fjc.0000166239.41830.3f 15838276

[98] Zanatta CM Veronese FV Loreto Mda S Sortica DA Carpio VN Eldeweiss MI. Endothelin-1 and endothelin a receptor immunoreactivity is increased in patients with diabetic nephropathy. Ren Fail. (2012) 34:308–15. doi: 10.3109/0886022x.2011.647301 22250646

[99] Asanuma K Mundel P. The role of podocytes in glomerular pathobiology. Clin Exp Nephrol. (2003) 7:255–9. doi: 10.1007/s10157-003-0259-6 14712353

[100] Morigi M Buelli S Angioletti S Zanchi C Longaretti L Zoja C. In response to protein load podocytes reorganize cytoskeleton and modulate endothelin-1 gene: implication for permselective dysfunction of chronic nephropathies. Am J Pathol. (2005) 166:1309–20. doi: 10.1016/s0002-9440(10)62350-4 PMC1606387 15855633

[101] Buelli S Rosanò L Gagliardini E Corna D Longaretti L Pezzotta A. β-arrestin-1 drives endothelin-1-mediated podocyte activation and sustains renal injury. J Am Soc Nephrol. (2014) 25:523–33. doi: 10.1681/asn.2013040362 PMC3935587 24371298

[102] Daehn I Casalena G Zhang T Shi S Fenninger F Barasch N. Endothelial mitochondrial oxidative stress determines podocyte depletion in segmental glomerulosclerosis. J Clin Invest. (2014) 124:1608–21. doi: 10.1172/jci71195 PMC3973074 24590287

[103] Ebefors K Wiener RJ Yu L Azeloglu EU Yi Z. Endothelin receptor-A mediates degradation of the glomerular endothelial surface layer via pathologic crosstalk between activated podocytes and glomerular endothelial cells. Kidney Int. (2019) 96:957–70. doi: 10.1016/j.kint.2019.05.007 PMC7200072 31402170

[104] Garg SS Gupta J. Polyol pathway and redox balance in diabetes. Pharmacol Res. (2022) 182:106326. doi: 10.1016/j.phrs.2022.106326 35752357

[105] Dunlop M. Aldose reductase and the role of the polyol pathway in diabetic nephropathy. Kidney Int Suppl. (2000) 77:S3–12. doi: 10.1046/j.1523-1755.2000.07702.x 10997684

[106] Cohen MP Klepser H. Glomerular Na+-K+-ATPase activity in acute and chronic diabetes and with aldose reductase inhibition. Diabetes. (1988) 37:558–62. doi: 10.2337/diab.37.5.558 2834250

[107] Yamaoka T Nishimura C Yamashita K Itakura M Yamada T Fujimoto J. Acute onset of diabetic pathological changes in transgenic mice with human aldose reductase cDNA. Diabetologia. (1995) 38:255–61. doi: 10.1007/bf00400627 7758869

[108] Sánchez-Lozada LG Tapia E Jiménez A Bautista P Cristóbal M Nepomuceno T. Fructose-induced metabolic syndrome is associated with glomerular hypertension and renal microvascular damage in rats. Am J Physiol Renal Physiol. (2007) 292:F423–429. doi: 10.1152/ajprenal.00124.2006 16940562

[109] Averill-Bates DA. The antioxidant glutathione. Vitam Horm. (2023) 121:109–41. doi: 10.1016/bs.vh.2022.09.002 36707132

[110] Tsugawa T Shinohara R Nagasaka A Nakano I Takeda F Nagata M. Alteration of urinary sorbitol excretion in WBN-kob diabetic rats - treatment with an aldose reductase inhibitor. J Endocrinol. (2004) 181:429–35. doi: 10.1677/joe.0.1810429 15171691

[111] Bucala R Vlassara H. Advanced glycosylation end products in diabetic renal and vascular disease. Am J Kidney Dis. (1995) 26:875–88. doi: 10.1016/0272-6386(95)90051-9 7503061

[112] Singh R Barden A Mori T Beilin L. Advanced glycation end-products: a review. Diabetologia. (2001) 44:129–46. doi: 10.1007/s001250051591 11270668

[113] Parwani K Mandal P. Role of advanced glycation end products and insulin resistance in diabetic nephropathy. Arch Physiol Biochem. (2023) 129:95–107. doi: 10.1080/13813455.2020.1797106 32730131

[114] An XF Zhou L Jiang PJ Yan M Huang YJ Zhang SN. Advanced glycation end-products induce heparanase expression in endothelial cells by the receptor for advanced glycation end products and through activation of the FOXO4 transcription factor. Mol Cell Biochem. (2011) 354:47–55. doi: 10.1007/s11010-011-0804-7 21461610

[115] Liu H Wang L Weng X Chen H Du Y Diao C. Inhibition of Brd4 alleviates renal ischemia/reperfusion injury-induced apoptosis and endoplasmic reticulum stress by blocking FoxO4-mediated oxidative stress. Redox Biol. (2019) 24:101195. doi: 10.1016/j.redox.2019.101195 31004990 PMC6475721

[116] Shu A Du Q Chen J Gao Y Zhu Y Lv G. Catalpol ameliorates endothelial dysfunction and inflammation in diabetic nephropathy via suppression of RAGE/RhoA/ROCK signaling pathway. Chem Biol Interact. (2021) 348:109625. doi: 10.1016/j.cbi.2021.109625 34416245

[117] Shen C Li Q Zhang YC Ma G Feng Y Zhu Q. Advanced glycation endproducts increase EPC apoptosis and decrease nitric oxide release via MAPK pathways. BioMed Pharmacother. (2010) 64:35–43. doi: 10.1016/j.biopha.2009.03.002 19766439

[118] Talmor Y Golan E Benchetrit S Bernheim J Klein O Green J. Calcitriol blunts the deleterious impact of advanced glycation end products on endothelial cells. Am J Physiol Renal Physiol. (2008) 294:F1059–1064. doi: 10.1152/ajprenal.00051.2008 18353875

[119] Tan AL Sourris KC Harcourt BE Thallas-Bonke V Penfold S Andrikopoulos S. Disparate effects on renal and oxidative parameters following RAGE deletion, AGE accumulation inhibition, or dietary AGE control in experimental diabetic nephropathy. Am J Physiol Renal Physiol. (2010) 298:F763–770. doi: 10.1152/ajprenal.00591.2009 20015941

[120] Apte RS Chen DS Ferrara N. VEGF in signaling and disease: beyond discovery and development. Cell. (2019) 176:1248–64. doi: 10.1016/j.cell.2019.01.021 PMC6410740 30849371

[121] Pajusola K Aprelikova O Korhonen J Kaipainen A Pertovaara L Alitalo R. FLT4 receptor tyrosine kinase contains seven immunoglobulin-like loops and is expressed in multiple human tissues and cell lines. Cancer Res. (1992) 52:5738–43. 1327515

[122] Yang J Liu Z. Mechanistic pathogenesis of endothelial dysfunction in diabetic nephropathy and retinopathy. Front Endocrinol (Lausanne). (2022) 13:816400. doi: 10.3389/fendo.2022.816400 35692405 PMC9174994

[123] Lugano R Ramachandran M Dimberg A. Tumor angiogenesis: causes, consequences, challenges and opportunities. Cell Mol Life Sci. (2020) 77:1745–70. doi: 10.1007/s00018-019-03351-7 PMC7190605 31690961

[124] Flyvbjerg A Khatir DS Jensen LJ Dagnaes-Hansen F Gronbaek H Rasch R. The involvement of growth hormone (GH), insulin-like growth factors (IGFs) and vascular endothelial growth factor (VEGF) in diabetic kidney disease. Curr Pharm Des. (2004) 10:3385–94. doi: 10.2174/1381612043383106 15544523

[125] Murakami H Tamasawa N Matsui J Yamato K JingZhi G Suda T. Plasma levels of soluble vascular adhesion molecule-1 and cholesterol oxidation product in type 2 diabetic patients with nephropathy. J Atheroscler Thromb. (2001) 8:21–4. doi: 10.5551/jat1994.8.21 11686311

[126] Eremina V Sood M Haigh J Nagy A Lajoie G Ferrara N. Glomerular-specific alterations of VEGF-A expression lead to distinct congenital and acquired renal diseases. J Clin Invest. (2003) 111:707–16. doi: 10.1172/jci17423 PMC151905 12618525

[127] Eremina V Jefferson JA Kowalewska J Hochster H Haas M Weisstuch J. VEGF inhibition and renal thrombotic microangiopathy. N Engl J Med. (2008) 358:1129–36. doi: 10.1056/NEJMoa0707330 PMC3030578 18337603

[128] Brosius FC Coward RJ. Podocytes, signaling pathways, and vascular factors in diabetic kidney disease. Adv Chronic Kidney Dis. (2014) 21:304–10. doi: 10.1053/j.ackd.2014.03.011 PMC4075065 24780459

[129] Kim BS Goligorsky MS. Role of VEGF in kidney development, microvascular maintenance and pathophysiology of renal disease. Korean J Intern Med. (2003) 18:65–75. doi: 10.3904/kjim.2003.18.2.65 12872442 PMC4531610

[130] Sato W Kosugi T Zhang L Roncal CA Heinig M Campbell-Thompson M. The pivotal role of VEGF on glomerular macrophage infiltration in advanced diabetic nephropathy. Lab Invest. (2008) 88:949–61. doi: 10.1038/labinvest.2008.60 18607348

[131] Onions KL Gamez M Buckner NR Baker SL Betteridge KB Desideri S. VEGFC reduces glomerular albumin permeability and protects against alterations in VEGF receptor expression in diabetic nephropathy. Diabetes. (2019) 68:172–87. doi: 10.2337/db18-0045 30389746

[132] Liu D Ding Q Dai DF Padhy B Nayak MK Li C. Loss of diacylglycerol kinase ϵ causes thrombotic microangiopathy by impairing endothelial VEGFA signaling. JCI Insight. (2021) 6:e146959. doi: 10.1172/jci.insight.146959 33986189 PMC8262293

[133] Brewster UC Perazella MA. The renin-angiotensin-aldosterone system and the kidney: effects on kidney disease. Am J Med. (2004) 116:263–72. doi: 10.1016/j.amjmed.2003.09.034 14969655

[134] Lovshin JA Boulet G Lytvyn Y Lovblom LE Bjornstad P Farooqi MA. Renin-angiotensin-aldosterone system activation in long-standing type 1 diabetes. JCI Insight. (2018) 3:e96968. doi: 10.1172/jci.insight.96968 29321380 PMC5821172

[135] Casare FA Thieme K Costa-Pessoa JM Rossoni LV Couto GK Fernandes FB. Renovascular remodeling and renal injury after extended angiotensin II infusion. Am J Physiol Renal Physiol. (2016) 310:F1295–1307. doi: 10.1152/ajprenal.00471.2015 26962104

[136] Taguchi S Azushima K Yamaji T Suzuki T Abe E Tanaka S. Angiotensin II type 1 receptor-associated protein deletion combined with angiotensin II stimulation accelerates the development of diabetic kidney disease in mice on a C57BL/6 strain. Hypertens Res. (2024) 47:55–66. doi: 10.1038/s41440-023-01496-4 37957242

[137] Nalin N Al Dhanhani A AlBawardi A Sharma C Chandran S Yasin J. Effect of angiotensin II on diabetic glomerular hyperpermeability: an *in vivo* permeability study in rats. Am J Physiol Renal Physiol. (2020) 319:F833–f838. doi: 10.1152/ajprenal.00259.2020 33017190

[138] Mende CW Samarakoon R Higgins PJ. Mineralocorticoid receptor-associated mechanisms in diabetic kidney disease and clinical significance of mineralocorticoid receptor antagonists. Am J Nephrol. (2023) 54:50–61. doi: 10.1159/000528783 36682353 PMC10273909

[139] Bauersachs J Jaisser F Toto R. Mineralocorticoid receptor activation and mineralocorticoid receptor antagonist treatment in cardiac and renal diseases. Hypertension. (2015) 65:257–63. doi: 10.1161/hypertensionaha.114.04488 25368026

[140] Perkovic V Jardine MJ Neal B Bompoint S Heerspink HJL Charytan DM. Canagliflozin and renal outcomes in type 2 diabetes and nephropathy. N Engl J Med. (2019) 380:2295–306. doi: 10.1056/NEJMoa1811744 30990260

[141] Bakris GL Agarwal R Chan JC Cooper ME Gansevoort RT Haller H. Effect of finerenone on albuminuria in patients with diabetic nephropathy: A randomized clinical trial. JAMA. (2015) 314:884–94. doi: 10.1001/jama.2015.10081 26325557

[142] Otsuka H Abe M Kobayashi H. The effect of aldosterone on cardiorenal and metabolic systems. Int J Mol Sci. (2023) 24:5370. doi: 10.3390/ijms24065370 36982445 PMC10049192

[143] Gill PS Wilcox CS. NADPH oxidases in the kidney. Antioxid Redox Signal. (2006) 8:1597–607. doi: 10.1089/ars.2006.8.1597 16987014

[144] Zhang PN Zhou MQ Guo J Zheng HJ Tang J Zhang C. Mitochondrial dysfunction and diabetic nephropathy: nontraditional therapeutic opportunities. J Diabetes Res. (2021) 2021:1010268. doi: 10.1155/2021/1010268 34926696 PMC8677373

[145] Nagasu H Satoh M Kiyokage E Kidokoro K Toida K Channon KM. Activation of endothelial NAD(P)H oxidase accelerates early glomerular injury in diabetic mice. Lab Invest. (2016) 96:25–36. doi: 10.1038/labinvest.2015.128 26552047 PMC4874489

[146] Schmidt HM Kelley EE Straub AC. The impact of xanthine oxidase (XO) on hemolytic diseases. Redox Biol. (2019) 21:101072. doi: 10.1016/j.redox.2018.101072 30580157 PMC6305892

[147] Xu N Jiang S Persson PB Persson EAG Lai EY Patzak A. Reactive oxygen species in renal vascular function. Acta Physiol (Oxf). (2020) 229:e13477. doi: 10.1111/apha.13477 32311827

[148] Li L Lai EY Luo Z Solis G Griendling KK Taylor WR. Superoxide and hydrogen peroxide counterregulate myogenic contractions in renal afferent arterioles from a mouse model of chronic kidney disease. Kidney Int. (2017) 92:625–33. doi: 10.1016/j.kint.2017.02.009 PMC12825038 28396118

[149] Schoonmaker GC Fallet RW Carmines PK. Superoxide anion curbs nitric oxide modulation of afferent arteriolar ANG II responsiveness in diabetes mellitus. Am J Physiol Renal Physiol. (2000) 278:F302–309. doi: 10.1152/ajprenal.2000.278.2.F302 10662734

[150] Zhang S Huang Q Wang Q Wang Q Cao X Zhao L. Enhanced renal afferent arteriolar reactive oxygen species and contractility to endothelin-1 are associated with canonical wnt signaling in diabetic mice. Kidney Blood Press Res. (2018) 43:860–71. doi: 10.1159/000490334 PMC6050514 29870994

[151] Xu N Wang Q Jiang S Wang Q Hu W Zhou S. Fenofibrate improves vascular endothelial function and contractility in diabetic mice. Redox Biol. (2019) 20:87–97. doi: 10.1016/j.redox.2018.09.024 30296701 PMC6174921

[152] Satheesan A Kumar J Leela KV Murugesan R Chaithanya V Angelin M. Review on the role of nucleotide-binding oligomerization domain-like receptor protein 3 (NLRP3) inflammasome pathway in diabetes: mechanistic insights and therapeutic implications. Inflammopharmacology. (2024) 32:2753–79. doi: 10.1007/s10787-024-01556-2 39160391

[153] Shen J Dai Z Li Y Zhu H Zhao L. TLR9 regulates NLRP3 inflammasome activation via the NF-kB signaling pathway in diabetic nephropathy. Diabetol Metab Syndr. (2022) 14:26. doi: 10.1186/s13098-021-00780-y 35120573 PMC8815223

[154] Habas A Reddy Natala S Bowden-Verhoek JK Stocking EM Price DL Wrasidlo W. NPT1220-312, a TLR2/TLR9 small molecule antagonist, inhibits pro-inflammatory signaling, cytokine release, and NLRP3 inflammasome activation. Int J Inflam. (2022) 2022:2337363. doi: 10.1155/2022/2337363 35265316 PMC8898874

[155] Wang D Wang H Gao H Zhang H Zhang H Wang Q. P2X7 receptor mediates NLRP3 inflammasome activation in depression and diabetes. Cell Biosci. (2020) 10:28. doi: 10.1186/s13578-020-00388-1 32166013 PMC7059335

[156] Lee HM Kim JJ Kim HJ Shong M Ku BJ Jo EK. Upregulated NLRP3 inflammasome activation in patients with type 2 diabetes. Diabetes. (2013) 62:194–204. doi: 10.2337/db12-0420 23086037 PMC3526026

[157] Gupta N Sahu A Prabhakar A Chatterjee T Tyagi T Kumari B. Activation of NLRP3 inflammasome complex potentiates venous thrombosis in response to hypoxia. Proc Natl Acad Sci U S A. (2017) 114:4763–8. doi: 10.1073/pnas.1620458114 PMC5422823 28420787

[158] Li XX Chen ZD Sun XJ Yang YQ Jin H Liu NF. Empagliflozin ameliorates vascular calcification in diabetic mice through inhibiting Bhlhe40-dependent NLRP3 inflammasome activation. Acta Pharmacol Sin. (2024) 45:751–64. doi: 10.1038/s41401-023-01217-0 PMC10943241 38172306

[159] Chow FY Nikolic-Paterson DJ Ozols E Atkins RC Rollin BJ Tesch GH. Monocyte chemoattractant protein-1 promotes the development of diabetic renal injury in streptozotocin-treated mice. Kidney Int. (2006) 69:73–80. doi: 10.1038/sj.ki.5000014 16374426

[160] Chow F Ozols E Nikolic-Paterson DJ Atkins RC Tesch GH. Macrophages in mouse type 2 diabetic nephropathy: correlation with diabetic state and progressive renal injury. Kidney Int. (2004) 65:116–28. doi: 10.1111/j.1523-1755.2004.00367.x 14675042

[161] Schroder K Zhou R Tschopp J. The NLRP3 inflammasome: a sensor for metabolic danger? Science. (2010) 327:296–300. doi: 10.1126/science.1184003 20075245

[162] Tschopp J Schroder K. NLRP3 inflammasome activation: The convergence of multiple signalling pathways on ROS production? Nat Rev Immunol. (2010) 10:210–5. doi: 10.1038/nri2725 20168318

[163] Shahzad K Bock F Dong W Wang H Kopf S Kohli S. Nlrp3-inflammasome activation in non-myeloid-derived cells aggravates diabetic nephropathy. Kidney Int. (2015) 87:74–84. doi: 10.1038/ki.2014.271 25075770 PMC4284813

[164] Qiu YY Tang LQ. Roles of the NLRP3 inflammasome in the pathogenesis of diabetic nephropathy. Pharmacol Res. (2016) 114:251–64. doi: 10.1016/j.phrs.2016.11.004 27826011

[165] Shahzad K Fatima S Khawaja H Elwakiel A Gadi I Ambreen S. Podocyte-specific Nlrp3 inflammasome activation promotes diabetic kidney disease. Kidney Int. (2022) 102:766–79. doi: 10.1016/j.kint.2022.06.010 35779608

[166] Gao P Meng XF Su H He FF Chen S Tang H. Thioredoxin-interacting protein mediates NALP3 inflammasome activation in podocytes during diabetic nephropathy. Biochim Biophys Acta. (2014) 1843:2448–60. doi: 10.1016/j.bbamcr.2014.07.001 25017793

[167] Zhao M Bai M Ding G Zhang Y Huang S Jia Z. Angiotensin II stimulates the NLRP3 inflammasome to induce podocyte injury and mitochondrial dysfunction. Kidney Dis (Basel). (2018) 4:83–94. doi: 10.1159/000488242 29998123 PMC6029226

[168] Wu M Han W Song S Du Y Liu C Chen N. NLRP3 deficiency ameliorates renal inflammation and fibrosis in diabetic mice. Mol Cell Endocrinol. (2018) 478:115–25. doi: 10.1016/j.mce.2018.08.002 30098377

[169] Lin J Cheng A Cheng K Deng Q Zhang S Lan Z. New insights into the mechanisms of pyroptosis and implications for diabetic kidney disease. Int J Mol Sci. (2020) 21:7057. doi: 10.3390/ijms21197057 32992874 PMC7583981

[170] Zhang S Guo S Wang P Song Y Yang L Sun Q. Dapagliflozin attenuates skeletal muscle atrophy in diabetic nephropathy mice through suppressing Gasdermin D-mediated pyroptosis. Int Immunopharmacol. (2025) 148:114088. doi: 10.1016/j.intimp.2025.114088 39837016

[171] Hu Z Zhou Y Gao C Liu J Pan C Guo J. Astragaloside IV attenuates podocyte apoptosis via regulating TXNIP/NLRP3/GSDMD signaling pathway in diabetic nephropathy. Diabetol Metab Syndr. (2024) 16:296. doi: 10.1186/s13098-024-01546-y 39696607 PMC11656976

[172] Kong X Zhao Y Wang X Yu Y Meng Y Yan G. Loganin reduces diabetic kidney injury by inhibiting the activation of NLRP3 inflammasome-mediated pyroptosis. Chem Biol Interact. (2023) 382:110640. doi: 10.1016/j.cbi.2023.110640 37473909

[173] Zhang WJ Chen SJ Zhou SC Wu SZ Wang H. Inflammasomes and fibrosis. Front Immunol. (2021) 12:643149. doi: 10.3389/fimmu.2021.643149 34177893 PMC8226128

[174] Soares JLS Fernandes FP Patente TA Monteiro MB Parisi MC Giannella-Neto D. Gain-of-function variants in NLRP1 protect against the development of diabetic kidney disease: NLRP1 inflammasome role in metabolic stress sensing? Clin Immunol. (2018) 187:46–9. doi: 10.1016/j.clim.2017.10.003 29031829

[175] Dai Y Zhou J Shi C. Inflammasome: structure, biological functions, and therapeutic targets. MedComm (2020). (2023) 4:e391. doi: 10.1002/mco2.391 37817895 PMC10560975

[176] Yuan F Kolb R Pandey G Li W Sun L Liu F. Involvement of the NLRC4-inflammasome in diabetic nephropathy. PloS One. (2016) 11:e0164135. doi: 10.1371/journal.pone.0164135 27706238 PMC5051905

[177] Yang H Xie T Li D Du X Wang T Li C. Tim-3 aggravates podocyte injury in diabetic nephropathy by promoting macrophage activation via the NF-κB/TNF-α pathway. Mol Metab. (2019) 23:24–36. doi: 10.1016/j.molmet.2019.02.007 30862474 PMC6479760

[178] Dong W Luo M Li Y Chen X Li L Chang Q. MICT ameliorates hypertensive nephropathy by inhibiting TLR4/NF-κB pathway and down-regulating NLRC4 inflammasome. PloS One. (2024) 19:e0306137. doi: 10.1371/journal.pone.0306137 39052650 PMC11271930

[179] Komada T Chung H Lau A Platnich JM Beck PL Benediktsson H. Macrophage uptake of necrotic cell DNA activates the AIM2 inflammasome to regulate a proinflammatory phenotype in CKD. J Am Soc Nephrol. (2018) 29:1165–81. doi: 10.1681/asn.2017080863 PMC5875955 29439156

[180] Li L Zhang L Cai Y Li J Zheng S Wang W. DNA damage-induced AIM2 pyroptosis in high glucose-induced proximal tubular epithelial cell. Front Cell Dev Biol. (2024) 12:1457369. doi: 10.3389/fcell.2024.1457369 39659523 PMC11628503

[181] Liu Y Zhang M Zeng L Lai Y Wu S Su X. Wogonin upregulates SOCS3 to alleviate the injury in Diabetic Nephropathy by inhibiting TLR4-mediated JAK/STAT/AIM2 signaling pathway. Mol Med. (2024) 30:78. doi: 10.1186/s10020-024-00845-4 38844873 PMC11155057

[182] Pandey A Li Z Gautam M Ghosh A Man SM. Molecular mechanisms of emerging inflammasome complexes and their activation and signaling in inflammation and pyroptosis. Immunol Rev. (2025) 329:e13406. doi: 10.1111/imr.13406 39351983 PMC11742652

[183] Grenier JM Wang L Manji GA Huang WJ Al-Garawi A Kelly R. Functional screening of five PYPAF family members identifies PYPAF5 as a novel regulator of NF-kappaB and caspase-1. FEBS Lett. (2002) 530:73–8. doi: 10.1016/s0014-5793(02)03416-6 12387869

[184] Valiño-Rivas L Cuarental L Nuñez G Sanz AB Ortiz A Sanchez-Niño MD. Loss of NLRP6 expression increases the severity of acute kidney injury. Nephrol Dial Transplant. (2020) 35:587–98. doi: 10.1093/ndt/gfz169 31504777

[185] Valiño-Rivas L Pintor-Chocano A Carriazo SM Sanz AB Ortiz A Sanchez-Niño MD. Loss of NLRP6 increases the severity of kidney fibrosis. J Cell Physiol. (2024) 239:e31347. doi: 10.1002/jcp.31347 38934623

[186] Teng B Duong M Tossidou I Yu X Schiffer M. Role of protein kinase C in podocytes and development of glomerular damage in diabetic nephropathy. Front Endocrinol (Lausanne). (2014) 5:179. doi: 10.3389/fendo.2014.00179 25414693 PMC4220730

[187] Takenaka T Forster H Epstein M. Protein kinase C and calcium channel activation as determinants of renal vasoconstriction by angiotensin II and endothelin. Circ Res. (1993) 73:743–50. doi: 10.1161/01.res.73.4.743 8396506

[188] Kuboki K Jiang ZY Takahara N Ha SW Igarashi M Yamauchi T. Regulation of endothelial constitutive nitric oxide synthase gene expression in endothelial cells and *in vivo*: a specific vascular action of insulin. Circulation. (2000) 101:676–81. doi: 10.1161/01.cir.101.6.676 10673261

[189] Williams B Gallacher B Patel H Orme C. Glucose-induced protein kinase C activation regulates vascular permeability factor mRNA expression and peptide production by human vascular smooth muscle cells *in vitro* . Diabetes. (1997) 46:1497–503. doi: 10.2337/diab.46.9.1497 9287052

[190] Craven PA Studer RK Felder J Phillips S DeRubertis FR. Nitric oxide inhibition of transforming growth factor-beta and collagen synthesis in mesangial cells. Diabetes. (1997) 46:671–81. doi: 10.2337/diab.46.4.671 9075810

[191] Matrougui K. Diabetes and microvascular pathophysiology: role of epidermal growth factor receptor tyrosine kinase. Diabetes Metab Res Rev. (2010) 26:13–6. doi: 10.1002/dmrr.1050 PMC2823570 19943320

[192] Zeng F Singh AB Harris RC. The role of the EGF family of ligands and receptors in renal development, physiology and pathophysiology. Exp Cell Res. (2009) 315:602–10. doi: 10.1016/j.yexcr.2008.08.005 PMC2654782 18761338

[193] Harris RC. The Role of the Epidermal Growth Factor Receptor in Diabetic Kidney Disease. Cells. (2022) 11:3416. doi: 10.3390/cells11213416 36359813 PMC9656309

[194] Helle F Jouzel C Chadjichristos C Placier S Flamant M Guerrot D. Improvement of renal hemodynamics during hypertension-induced chronic renal disease: role of EGF receptor antagonism. Am J Physiol Renal Physiol. (2009) 297:F191–199. doi: 10.1152/ajprenal.00015.2009 19420116

[195] Palen DI Matrougui K. Role of elevated EGFR phosphorylation in the induction of structural remodelling and altered mechanical properties of resistance artery from type 2 diabetic mice. Diabetes Metab Res Rev. (2008) 24:651–6. doi: 10.1002/dmrr.905 PMC2720308 18973206

[196] Akhtar S Chandrasekhar B Yousif MH Renno W Benter IF El-Hashim AZ. Chronic administration of nano-sized PAMAM dendrimers *in vivo* inhibits EGFR-ERK1/2-ROCK signaling pathway and attenuates diabetes-induced vascular remodeling and dysfunction. Nanomedicine. (2019) 18:78–89. doi: 10.1016/j.nano.2019.02.012 30844576

[197] Schreier B Stern C Dubourg V Nolze A Rabe S Mildenberger S. Endothelial epidermal growth factor receptor is of minor importance for vascular and renal function and obesity-induced dysfunction in mice. Sci Rep. (2021) 11:7269. doi: 10.1038/s41598-021-86587-3 33790318 PMC8012653

[198] Cao Z Liu Y Wang Y Leng P. Research progress on the role of PDGF/PDGFR in type 2 diabetes. BioMed Pharmacother. (2023) 164:114983. doi: 10.1016/j.biopha.2023.114983 37290188

[199] Jung SC Kang D Ko EA. Roles of PDGF/PDGFR signaling in various organs. Korean J Physiol Pharmacol. (2024) 29:139–55. doi: 10.4196/kjpp.24.309 PMC11842291 39482238

[200] Ma CN Shi SR Zhang XY Xin GS Zou X Li WL. Targeting PDGF/PDGFR Signaling Pathway by microRNA, lncRNA, and circRNA for Therapy of Vascular Diseases: A Narrow Review. Biomolecules. (2024) 14:1446. doi: 10.3390/biom14111446 39595622 PMC11592287

[201] Yasunari K Kohno M Kano H Yokokawa K Minami M Yoshikawa J. Dopamine D1-like receptor stimulation inhibits hypertrophy induced by platelet-derived growth factor in cultured rat renal vascular smooth muscle cells. Hypertension. (1997) 29:350–5. doi: 10.1161/01.hyp.29.1.350 9039126

[202] Langham RG Kelly DJ Maguire J Dowling JP Gilbert RE Thomson NM. Over-expression of platelet-derived growth factor in human diabetic nephropathy. Nephrol Dial Transplant. (2003) 18:1392–6. doi: 10.1093/ndt/gfg177 12808179

[203] Boor P Bábíčková J Steegh F Hautvast P Martin IV Djudjaj S. Role of platelet-derived growth factor-CC in capillary rarefaction in renal fibrosis. Am J Pathol. (2015) 185:2132–42. doi: 10.1016/j.ajpath.2015.04.022 26216283

[204] Floege J Eitner F Alpers CE. A new look at platelet-derived growth factor in renal disease. J Am Soc Nephrol. (2008) 19:12–23. doi: 10.1681/asn.2007050532 18077793

[205] Das F Ghosh-Choudhury N Venkatesan B Kasinath BS Ghosh Choudhury G. PDGF receptor-β uses Akt/mTORC1 signaling node to promote high glucose-induced renal proximal tubular cell collagen I (α2) expression. Am J Physiol Renal Physiol. (2017) 313:F291–f307. doi: 10.1152/ajprenal.00666.2016 28424212 PMC5582895

[206] Noshahr ZS Salmani H Khajavi Rad A Sahebkar A. Animal models of diabetes-associated renal injury. J Diabetes Res. (2020) 2020:9416419. doi: 10.1155/2020/9416419 32566684 PMC7256713

[207] Kim NH Hyeon JS Kim NH Cho A Lee G Jang SY. Metabolic changes in urine and serum during progression of diabetic kidney disease in a mouse model. Arch Biochem Biophys. (2018) 646:90–7. doi: 10.1016/j.abb.2018.03.042 29621522

[208] Lorberbaum DS Sarbaugh D Sussel L. Leveraging the strengths of mice, human stem cells, and organoids to model pancreas development and diabetes. Front Endocrinol (Lausanne). (2022) 13:1042611. doi: 10.3389/fendo.2022.1042611 36339450 PMC9634409

[209] Kern TS Engerman RL. Arrest of glomerulopathy in diabetic dogs by improved glycaemic control. Diabetologia. (1990) 33:522–5. doi: 10.1007/bf00404138 2253827

[210] Qi Z Fujita H Jin J Davis LS Wang Y Fogo AB. Characterization of susceptibility of inbred mouse strains to diabetic nephropathy. Diabetes. (2005) 54:2628–37. doi: 10.2337/diabetes.54.9.2628 16123351

[211] Uchino H Kim T Lam TK Yoshii H Klement P Williams W. FK-614, a selective peroxisome proliferator-activated receptor gamma agonist, improves peripheral glucose utilization while decreasing hepatic insulin extraction in alloxan-induced diabetic dogs. Metabolism. (2005) 54:1250–8. doi: 10.1016/j.metabol.2005.04.012 16125538

[212] Wang J Takeuchi T Tanaka S Kubo SK Kayo T Lu D. A mutation in the insulin 2 gene induces diabetes with severe pancreatic beta-cell dysfunction in the Mody mouse. J Clin Invest. (1999) 103:27–37. doi: 10.1172/jci4431 9884331 PMC407861

[213] Yau C Danska JS. Cracking the type 1 diabetes code: Genes, microbes, immunity, and the early life environment. Immunol Rev. (2024) 325:23–45. doi: 10.1111/imr.13362 39166298

[214] Wang W Jiang S Tang X Cai L Epstein PN Cheng Y. Sex differences in progression of diabetic nephropathy in OVE26 type 1 diabetic mice. Biochim Biophys Acta Mol Basis Dis. (2020) 1866:165589. doi: 10.1016/j.bbadis.2019.165589 31678163

[215] Bortell R Yang C. The BB rat as a model of human type 1 diabetes. Methods Mol Biol. (2012) 933:31–44. doi: 10.1007/978-1-62703-068-7_3 22893399

[216] Tesch GH Allen TJ. Rodent models of streptozotocin-induced diabetic nephropathy. Nephrol (Carlton). (2007) 12:261–6. doi: 10.1111/j.1440-1797.2007.00796.x 17498121

[217] Liang J Liu Y. Animal models of kidney disease: challenges and perspectives. Kidney360. (2023) 4:1479–93. doi: 10.34067/kid.0000000000000227 PMC10617803 37526653

[218] Deji N Kume S Araki S Soumura M Sugimoto T Isshiki K. Structural and functional changes in the kidneys of high-fat diet-induced obese mice. Am J Physiol Renal Physiol. (2009) 296:F118–126. doi: 10.1152/ajprenal.00110.2008 18971213

[219] Ito T Tanimoto M Yamada K Kaneko S Matsumoto M Obayashi K. Glomerular changes in the KK-Ay/Ta mouse: a possible model for human type 2 diabetic nephropathy. Nephrol (Carlton). (2006) 11:29–35. doi: 10.1111/j.1440-1797.2006.00543.x 16509929

[220] Hirata T Yoshitomi T Inoue M Iigo Y Matsumoto K Kubota K. Pathological and gene expression analysis of a polygenic diabetes model, NONcNZO10/LtJ mice. Gene. (2017) 629:52–8. doi: 10.1016/j.gene.2017.07.075 28760554

[221] Nagao M Asai A Eliasson L Oikawa S. Selectively bred rodent models for studying the etiology of type 2 diabetes: Goto-Kakizaki rats and Oikawa-Nagao mice. Endocr J. (2023) 70:19–30. doi: 10.1507/endocrj.EJ22-0253 36477370

[222] Niibo M Kanasaki A Iida T Ohnishi K Ozaki T Akimitsu K. d-allulose protects against diabetic nephropathy progression in Otsuka Long-Evans Tokushima Fatty rats with type 2 diabetes. PloS One. (2022) 17:e0263300. doi: 10.1371/journal.pone.0263300 35100325 PMC8803202

[223] Liu G Shea CM Jones JE Price GM Warren W Lonie E. Praliciguat inhibits progression of diabetic nephropathy in ZSF1 rats and suppresses inflammation and apoptosis in human renal proximal tubular cells. Am J Physiol Renal Physiol. (2020) 319:F697–f711. doi: 10.1152/ajprenal.00003.2020 32865013

[224] Wada Y Kidokoro K Kondo M Tokuyama A Kadoya H Nagasu H. Evaluation of glomerular hemodynamic changes by sodium-glucose-transporter 2 inhibition in type 2 diabetic rats using *in vivo* imaging. Kidney Int. (2024) 106:408–18. doi: 10.1016/j.kint.2024.05.006 38801992

[225] Pérez Gutierrez RM García Campoy AH Paredes Carrera SP Muñiz Ramirez A Mota Flores JM Flores Valle SO. 3’-O-β-d-glucopyranosyl-α,4,2’,4’,6’-pentahydroxy-dihydrochalcone, from Bark of Eysenhardtia polystachya Prevents Diabetic Nephropathy via Inhibiting Protein Glycation in STZ-Nicotinamide Induced Diabetic Mice. Molecules. (2019) 24:1214. doi: 10.3390/molecules24071214 30925713 PMC6480600

[226] Shevalye H Lupachyk S Watcho P Stavniichuk R Khazim K Abboud HE. Prediabetic nephropathy as an early consequence of the high-calorie/high-fat diet: relation to oxidative stress. Endocrinology. (2012) 153:1152–61. doi: 10.1210/en.2011-1997 PMC3281531 22234462

[227] Xu H Ma Z Lu S Li R Lyu L Ding L. Renal resistive index as a novel indicator for renal complications in high-fat diet-fed mice. Kidney Blood Press Res. (2017) 42:1128–40. doi: 10.1159/000485781 29224015

[228] Kim DH Choi BH Ku SK Park JH Oh E Kwak MK. Beneficial effects of sarpogrelate and rosuvastatin in high fat diet/streptozotocin-induced nephropathy in mice. PLoS One. (2016) 11:e0153965. doi: 10.1371/journal.pone.0153965 27097221 PMC4838298

[229] Morris SM Jr. Gao T Cooper TK Kepka-Lenhart D Awad AS. Arginase-2 mediates diabetic renal injury. Diabetes. (2011) 60:3015–22. doi: 10.2337/db11-0901 PMC3198072 21926276

[230] Hudkins KL Pichaiwong W Wietecha T Kowalewska J Banas MC Spencer MW. BTBR Ob/Ob mutant mice model progressive diabetic nephropathy. J Am Soc Nephrol. (2010) 21:1533–42. doi: 10.1681/asn.2009121290 PMC3013527 20634301

[231] Ericsson A Tonelius P Lal M Sabirsh A Böttcher G William-Olsson L. The effects of dual PPARα/γ agonism compared with ACE inhibition in the BTBRob/ob mouse model of diabetes and diabetic nephropathy. Physiol Rep. (2017) 5:e13186. doi: 10.14814/phy2.13186 28292877 PMC5350186

[232] Westergren HU Grönros J Heinonen SE Miliotis T Jennbacken K Sabirsh A. Impaired coronary and renal vascular function in spontaneously type 2 diabetic leptin-deficient mice. PLoS One. (2015) 10:e0130648. doi: 10.1371/journal.pone.0130648 26098416 PMC4476758

[233] Feng X Wang S Sun Z Dong H Yu H Huang M. Ferroptosis Enhanced Diabetic Renal Tubular Injury via HIF-1α/HO-1 Pathway in db/db Mice. Front Endocrinol (Lausanne). (2021) 12:626390. doi: 10.3389/fendo.2021.626390 33679620 PMC7930496

[234] Jiang Y Xie F Lv X Wang S Liao X Yu Y. Mefunidone ameliorates diabetic kidney disease in STZ and db/db mice. FASEB J. (2021) 35:e21198. doi: 10.1096/fj.202001138RR 33225469

[235] Park S Bivona BJ Feng Y Lazartigues E Harrison-Bernard LM. Intact renal afferent arteriolar autoregulatory responsiveness in db/db mice. Am J Physiol Renal Physiol. (2008) 295:F1504–1511. doi: 10.1152/ajprenal.90417.2008 PMC2584903 18753291

[236] Yang G Zhao Z Zhang X Wu A Huang Y Miao Y. Effect of berberine on the renal tubular epithelial-to-mesenchymal transition by inhibition of the Notch/snail pathway in diabetic nephropathy model KKAy mice. Drug Des Devel Ther. (2017) 11:1065–79. doi: 10.2147/dddt.S124971 PMC5384688 28408805

[237] Ma Y Li W Yazdizadeh Shotorbani P Dubansky BH Huang L Chaudhari S. Comparison of diabetic nephropathy between male and female eNOS(-/-)db/db mice. Am J Physiol Renal Physiol. (2019) 316:F889–f897. doi: 10.1152/ajprenal.00023.2019 30810354 PMC6580249

[238] Wang W Mitra A Poole B Falk S Lucia MS Tayal S. Endothelial nitric oxide synthase-deficient mice exhibit increased susceptibility to endotoxin-induced acute renal failure. Am J Physiol Renal Physiol. (2004) 287:F1044–1048. doi: 10.1152/ajprenal.00136.2004 15475535

[239] Soler MJ Riera M Batlle D. New experimental models of diabetic nephropathy in mice models of type 2 diabetes: efforts to replicate human nephropathy. Exp Diabetes Res. (2012) 2012:616313. doi: 10.1155/2012/616313 22461787 PMC3291115

[240] Olsen AS Sarras MP Jr. Intine RV. Limb regeneration is impaired in an adult zebrafish model of diabetes mellitus. Wound Repair Regener. (2010) 18:532–42. doi: 10.1111/j.1524-475X.2010.00613.x PMC2941236 20840523

[241] Zhao Q Li J Yan J Liu S Guo Y Chen D. Lycium barbarum polysaccharides ameliorates renal injury and inflammatory reaction in alloxan-induced diabetic nephropathy rabbits. Life Sci. (2016) 157:82–90. doi: 10.1016/j.lfs.2016.05.045 27262790

[242] Wang R Lin Z Yang X Zhao K Wang S Sui X. Noninvasive evaluation of renal hypoxia by multiparametric functional MRI in early diabetic kidney disease. J Magn Reson Imaging. (2022) 55:518–27. doi: 10.1002/jmri.27814 34184356

[243] Phillips AO Baboolal K Riley S Gröne H Janssen U Steadman R. Association of prolonged hyperglycemia with glomerular hypertrophy and renal basement membrane thickening in the Goto Kakizaki model of non-insulin-dependent diabetes mellitus. Am J Kidney Dis. (2001) 37:400–10. doi: 10.1053/ajkd.2001.21322 11157383

[244] Vesely DL Gower WR Jr Dietz JR Overton RM Clark LC Antwi EK. Elevated atrial natriuretic peptides and early renal failure in type 2 diabetic Goto-Kakizaki rats. Metabolism. (1999) 48:771–8. doi: 10.1016/s0026-0495(99)90178-6 10381153

[245] Liu B Liang F Gu LP Wang CQ Li XH Jiang YM. Renal blood perfusion in GK rats using targeted contrast enhanced ultrasonography. Asian Pac J Trop Med. (2015) 8:668–73. doi: 10.1016/j.apjtm.2015.07.011 26321523

[246] Singh R Farooq SA Mannan A Singh TG Najda A Grażyna Z. Animal models of diabetic microvascular complications: Relevance to clinical features. BioMed Pharmacother. (2022) 145:112305. doi: 10.1016/j.biopha.2021.112305 34872802

[247] Hashimoto S Yamada K Kawata T Mochizuki T Schnermann J Koike T. Abnormal autoregulation and tubuloglomerular feedback in prediabetic and diabetic OLETF rats. Am J Physiol Renal Physiol. (2009) 296:F598–604. doi: 10.1152/ajprenal.00074.2008 19106213

[248] Kawano K Mori S Hirashima T Man ZW Natori T. Examination of the pathogenesis of diabetic nephropathy in OLETF rats. J Vet Med Sci. (1999) 61:1219–28. doi: 10.1292/jvms.61.1219 10593580

[249] Wang Z Liu Q Dai W Hua B Li H Li W. Pioglitazone downregulates Twist-1 expression in the kidney and protects renal function of Zucker diabetic fatty rats. BioMed Pharmacother. (2019) 118:109346. doi: 10.1016/j.biopha.2019.109346 31506251

[250] Zhang X Jia Y Jackson EK Tofovic SP. 2-Methoxyestradiol and 2-ethoxyestradiol retard the progression of renal disease in aged, obese, diabetic ZSF1 rats. J Cardiovasc Pharmacol. (2007) 49:56–63. doi: 10.1097/FJC.0b013e31802cb88e 17261964

[251] Sugano M Sugano M Yamato H Hayashi T Ochiai H Kakuchi J Goto S. High-fat diet in low-dose-streptozotocin-treated heminephrectomized rats induces all features of human type 2 diabetic nephropathy: a new rat model of diabetic nephropathy. Nutr Metab Cardiovasc Dis. (2006) 16:477–84. doi: 10.1016/j.numecd.2005.08.007 17015185

[252] Yao H Feng J Zheng Q Wei Y Yang G Feng W. Comparison of the effects of prophylactic and therapeutic administrations on peripheral neuropathy in streptozotocin-diabetic rats with gliclazide or methylcobalamin. Exp Clin Endocrinol Diabetes. (2020) 128:635–43. doi: 10.1055/a-0635-0672 30453342

[253] Brown DM Steffes MW Thibert P Azar S Mauer SM. Glomerular manifestations of diabetes in the BB rat. Metabolism. (1983) 32:131–5. doi: 10.1016/s0026-0495(83)80026-2 6865762

[254] Chang JH Paik SY Mao L Eisner W Flannery PJ Wang L. Diabetic kidney disease in FVB/NJ Akita mice: temporal pattern of kidney injury and urinary nephrin excretion. PLoS One. (2012) 7:e33942. doi: 10.1371/journal.pone.0033942 22496773 PMC3319540

[255] Riera M Anguiano L Clotet S Roca-Ho H Rebull M Pascual J. Paricalcitol modulates ACE2 shedding and renal ADAM17 in NOD mice beyond proteinuria. Am J Physiol Renal Physiol. (2016) 310:F534–546. doi: 10.1152/ajprenal.00082.2015 26697977

[256] Thibodeau JF Holterman CE Burger D Read NC Reudelhuber TL Kennedy CR. A novel mouse model of advanced diabetic kidney disease. PLoS One. (2014) 9:e113459. doi: 10.1371/journal.pone.0113459 25514595 PMC4267730

[257] Kern TS Engerman RL. Renal hemodynamics in experimentally galactosemic dogs and diabetic dogs. Metabolism. (1991) 40:450–4. doi: 10.1016/0026-0495(91)90223-j 1902544

[258] Li Q Zhang K Hou L Liao J Zhang H Han Q. Endoplasmic reticulum stress contributes to pyroptosis through NF-κB/NLRP3 pathway in diabetic nephropathy. Life Sci. (2023) 322:121656. doi: 10.1016/j.lfs.2023.121656 37011874

[259] Suortti P Thomlinson W. Medical applications of synchrotron radiation. Phys Med Biol. (2003) 48:R1–35. doi: 10.1088/0031-9155/48/13/201 12884920

[260] Miya K Matsushita S Hyodo K Tokunaga C Sakamoto H Mizutani T. Renal contrast microangiography with synchrotron radiation: a novel method for visualizing structures within nephrons *in vivo* . Acta Radiol. (2017) 58:505–10. doi: 10.1177/0284185116658685 27439400

[261] Eppel GA Jacono DL Shirai M Umetani K Evans RG Pearson JT. Contrast angiography of the rat renal microcirculation *in vivo* using synchrotron radiation. Am J Physiol Renal Physiol. (2009) 296:F1023–1031. doi: 10.1152/ajprenal.90499.2008 19261740

[262] Li S Wang F Sun D. The renal microcirculation in chronic kidney disease: novel diagnostic methods and therapeutic perspectives. Cell Biosci. (2021) 11:90. doi: 10.1186/s13578-021-00606-4 34001267 PMC8130426

[263] Boerckel JD Mason DE McDermott AM Alsberg E. Microcomputed tomography: approaches and applications in bioengineering. Stem Cell Res Ther. (2014) 5:144. doi: 10.1186/scrt534 25689288 PMC4290379

[264] Wälchli T Bisschop J Miettinen A Ulmann-Schuler A Hintermüller C Meyer EP. Hierarchical imaging and computational analysis of three-dimensional vascular network architecture in the entire postnatal and adult mouse brain. Nat Protoc. (2021) 16:4564–610. doi: 10.1038/s41596-021-00587-1 34480130

[265] Deng L Tang H Qiang J Wang J Xiao S. Blood supply of early lung adenocarcinomas in mice and the tumor-supplying vessel relationship: A micro-CT angiography study. Cancer Prev Res (Phila). (2020) 13:989–96. doi: 10.1158/1940-6207.Capr-20-0036 32816806

[266] Cao Y Zhou Y Ni S Wu T Li P Liao S. Three dimensional quantification of microarchitecture and vessel regeneration by synchrotron radiation microcomputed tomography in a rat model of spinal cord injury. J Neurotrauma. (2017) 34:1187–99. doi: 10.1089/neu.2016.4697 27676128

[267] Borland SJ Behnsen J Ashton N Francis SE Brennan K Sherratt MJ. X-ray micro-computed tomography: an emerging technology to analyze vascular calcification in animal models. Int J Mol Sci. (2020) 21:4538. doi: 10.3390/ijms21124538 32630604 PMC7352990

[268] Ehling J Bábíčková J Gremse F Klinkhammer BM Baetke S Knuechel R. Quantitative micro-computed tomography imaging of vascular dysfunction in progressive kidney diseases. J Am Soc Nephrol. (2016) 27:520–32. doi: 10.1681/asn.2015020204 PMC4724942 26195818

[269] Yamanaka N Shimizu A. Role of glomerular endothelial damage in progressive renal disease. Kidney Blood Press Res. (1999) 22:13–20. doi: 10.1159/000025904 10352403

[270] Hohenstein B Braun A Amann KU Johnson RJ Hugo CP. A murine model of site-specific renal microvascular endothelial injury and thrombotic microangiopathy. Nephrol Dial Transplant. (2008) 23:1144–56. doi: 10.1093/ndt/gfm774 18045820

[271] Kusano T Takano H Kang D Nagahama K Aoki M Morita M. Endothelial cell injury in acute and chronic glomerular lesions in patients with IgA nephropathy. Hum Pathol. (2016) 49:135–44. doi: 10.1016/j.humpath.2015.10.013 26826420

[272] Wagner MC Rhodes G Wang E Pruthi V Arif E Saleem MA. Ischemic injury to kidney induces glomerular podocyte effacement and dissociation of slit diaphragm proteins Neph1 and ZO-1. J Biol Chem. (2008) 283:35579–89. doi: 10.1074/jbc.M805507200 PMC2602882 18922801

[273] Bábíčková J Klinkhammer BM Buhl EM Djudjaj S Hoss M Heymann F. Regardless of etiology, progressive renal disease causes ultrastructural and functional alterations of peritubular capillaries. Kidney Int. (2017) 91:70–85. doi: 10.1016/j.kint.2016.07.038 27678159

[274] Advani A Connelly KA Yuen DA Zhang Y Advani SL Trogadis J. Fluorescent microangiography is a novel and widely applicable technique for delineating the renal microvasculature. PLoS One. (2011) 6:e24695. doi: 10.1371/journal.pone.0024695 21984894 PMC3184963

[275] Kramann R Tanaka M Humphreys BD. Fluorescence microangiography for quantitative assessment of peritubular capillary changes after AKI in mice. J Am Soc Nephrol. (2014) 25:1924–31. doi: 10.1681/asn.2013101121 PMC4147987 24652794

[276] Liang Y Walczak P. Long term intravital single cell tracking under multiphoton microscopy. J Neurosci Methods. (2021) 349:109042. doi: 10.1016/j.jneumeth.2020.109042 33340557

[277] Schießl IM Hammer A Riquier-Brison A Peti-Peterdi J. Just look! Intravital microscopy as the best means to study kidney cell death dynamics. Semin Nephrol. (2016) 36:220–36. doi: 10.1016/j.semnephrol.2016.03.009 PMC5425152 27339387

[278] Ferrell N Sandoval RM Bian A Campos-Bilderback SB Molitoris BA Fissell WH. Shear stress is normalized in glomerular capillaries following ⅚ nephrectomy. Am J Physiol Renal Physiol. (2015) 308:F588–593. doi: 10.1152/ajprenal.00290.2014 PMC4360039 25587117

[279] Martins JR Haenni D Bugarski M Polesel M Schuh C Hall AM. Intravital kidney microscopy: entering a new era. Kidney Int. (2021) 100:527–35. doi: 10.1016/j.kint.2021.02.042 34015315

[280] Le Bihan D Breton E Lallemand D Grenier P Cabanis E Laval-Jeantet M. MR imaging of intravoxel incoherent motions: application to diffusion and perfusion in neurologic disorders. Radiology. (1986) 161:401–7. doi: 10.1148/radiology.161.2.3763909 3763909

[281] Le Bihan D. What can we see with IVIM MRI? Neuroimage. (2019) 187:56–67. doi: 10.1016/j.neuroimage.2017.12.062 29277647

[282] Dai H Zhao C Xiong Y He Q Su W Li J. Evaluation of contrast-induced acute kidney injury using IVIM and DKI MRI in a rat model of diabetic nephropathy. Insights Imaging. (2022) 13:110. doi: 10.1186/s13244-022-01249-w 35767196 PMC9243200

[283] Pruijm M Hofmann L Piskunowicz M Muller ME Zweiacker C Bassi I. Determinants of renal tissue oxygenation as measured with BOLD-MRI in chronic kidney disease and hypertension in humans. PLoS One. (2014) 9:e95895. doi: 10.1371/journal.pone.0095895 24760031 PMC3997480

[284] Prasad PV. Evaluation of intra-renal oxygenation by BOLD MRI. Nephron Clin Pract. (2006) 103:c58–65. doi: 10.1159/000090610 16543757

[285] Chen F Li S Sun D. Methods of blood oxygen level-dependent magnetic resonance imaging analysis for evaluating renal oxygenation. Kidney Blood Press Res. (2018) 43:378–88. doi: 10.1159/000488072 29539614

[286] Ehman RL. Magnetic resonance elastography: from invention to standard of care. Abdom Radiol (NY). (2022) 47:3028–36. doi: 10.1007/s00261-022-03597-z PMC9538645 35852570

[287] Han JH Ahn JH Kim JS. Magnetic resonance elastography for evaluation of renal parenchyma in chronic kidney disease: a pilot study. Radiol Med. (2020) 125:1209–15. doi: 10.1007/s11547-020-01210-1 32367323

[288] Shatil AS Kirpalani A Younus E Tyrrell PN Krizova A Yuen DA. Magnetic resonance elastography-derived stiffness predicts renal function loss and is associated with microvascular inflammation in kidney transplant recipients. Transplant Direct. (2022) 8:e1334. doi: 10.1097/txd.0000000000001334 35721457 PMC9197345

[289] Zhang X Zhu X Ferguson CM Jiang K Burningham T Lerman A. Magnetic resonance elastography can monitor changes in medullary stiffness in response to treatment in the swine ischemic kidney. Magma. (2018) 31:375–82. doi: 10.1007/s10334-017-0671-7 PMC5976551 29289980

[290] Warner L Yin M Glaser KJ Woollard JA Carrascal CA Korsmo MJ. Noninvasive *In vivo* assessment of renal tissue elasticity during graded renal ischemia using MR elastography. Invest Radiol. (2011) 46:509–14. doi: 10.1097/RLI.0b013e3182183a95 PMC3128234 21467945

[291] Brown RS Sun MRM Stillman IE Russell TL Rosas SE Wei JL. The utility of magnetic resonance imaging for noninvasive evaluation of diabetic nephropathy. Nephrol Dial Transplant. (2020) 35:970–8. doi: 10.1093/ndt/gfz066 PMC7282829 31329940

[292] Hernandez-Garcia L Lahiri A Schollenberger J. Recent progress in ASL. Neuroimage. (2019) 187:3–16. doi: 10.1016/j.neuroimage.2017.12.095 29305164 PMC6030511

[293] Dong J Yang L Su T Yang X Chen B Zhang J. Quantitative assessment of acute kidney injury by noninvasive arterial spin labeling perfusion MRI: a pilot study. Sci China Life Sci. (2013) 56:745–50. doi: 10.1007/s11427-013-4503-3 23740361

[294] Hueper K Gutberlet M Rong S Hartung D Mengel M Lu X. Acute kidney injury: arterial spin labeling to monitor renal perfusion impairment in mice-comparison with histopathologic results and renal function. Radiology. (2014) 270:117–24. doi: 10.1148/radiol.13130367 24023073

[295] Jokisch F Buchner A Schulz GB Grimm T Weinhold P Pfitzinger PL. Prospective evaluation of 4-D contrast-enhanced-ultrasound (CEUS) imaging in bladder tumors. Clin Hemorheol Microcirc. (2020) 74:1–12. doi: 10.3233/ch-199231 31743990

[296] Srivastava S Dhyani M Dighe M. Contrast-enhanced ultrasound (CEUS): applications from the kidneys to the bladder. Abdom Radiol (NY). (2024) 49:4092–112. doi: 10.1007/s00261-024-04388-4 38884782

[297] Chung YE Kim KW. Contrast-enhanced ultrasonography: advance and current status in abdominal imaging. Ultrasonography. (2015) 34:3–18. doi: 10.14366/usg.14034 25342120 PMC4282229

[298] Tingle SJ Connelly C Glover EK Stenberg B McNeill A Kourounis G. Contrast-enhanced ultrasound to assess kidney quality during ex situ normothermic machine perfusion. Transpl Int. (2025) 38:14268. doi: 10.3389/ti.2025.14268 40242325 PMC11999844

[299] Chihanga T Ma Q Nicholson JD Ruby HN Edelmann RE Devarajan P. NMR spectroscopy and electron microscopy identification of metabolic and ultrastructural changes to the kidney following ischemia-reperfusion injury. Am J Physiol Renal Physiol. (2018) 314:F154–f166. doi: 10.1152/ajprenal.00363.2017 28978534 PMC5866452

[300] Li S Wang Y Wang Z Chen L Zuo B Liu C. Enhanced renoprotective effect of GDNF-modified adipose-derived mesenchymal stem cells on renal interstitial fibrosis. Stem Cell Res Ther. (2021) 12:27. doi: 10.1186/s13287-020-02049-z 33413640 PMC7792009

[301] Molitoris BA Sandoval RM Wagner MC. Intravital multiphoton microscopy as a tool for studying renal physiology, pathophysiology and therapeutics. Front Physiol. (2022) 13:827280. doi: 10.3389/fphys.2022.827280 35399274 PMC8988037

[302] Sandoval RM Molitoris BA. Intravital multiphoton microscopy as a tool for studying renal physiology and pathophysiology. Methods. (2017) 128:20–32. doi: 10.1016/j.ymeth.2017.07.014 28733090 PMC5730351

[303] Schießl IM Castrop H. Deep insights: intravital imaging with two-photon microscopy. Pflugers Arch. (2016) 468:1505–16. doi: 10.1007/s00424-016-1832-7 27352273

[304] Zhang JL Lee VS. Renal perfusion imaging by MRI. J Magn Reson Imaging. (2020) 52:369–79. doi: 10.1002/jmri.26911 31452303

[305] Zimmer F Zöllner FG Hoeger S Klotz S Tsagogiorgas C Krämer BK. Quantitative renal perfusion measurements in a rat model of acute kidney injury at 3T: testing inter- and intramethodical significance of ASL and DCE-MRI. PLoS One. (2013) 8:e53849. doi: 10.1371/journal.pone.0053849 23308289 PMC3538736

[306] Jovic D Liang X Zeng H Lin L Xu F Luo Y. Single-cell RNA sequencing technologies and applications: A brief overview. Clin Transl Med. (2022) 12:e694. doi: 10.1002/ctm2.694 35352511 PMC8964935

[307] Dumas SJ Meta E Borri M Goveia J Rohlenova K Conchinha NV. Single-cell RNA sequencing reveals renal endothelium heterogeneity and metabolic adaptation to water deprivation. J Am Soc Nephrol. (2020) 31:118–38. doi: 10.1681/asn.2019080832 PMC6935008 31818909

[308] Fu J Akat KM Sun Z Zhang W Schlondorff D Liu Z. Single-cell RNA profiling of glomerular cells shows dynamic changes in experimental diabetic kidney disease. J Am Soc Nephrol. (2019) 30:533–45. doi: 10.1681/asn.2018090896 PMC6442341 30846559

[309] Zhang X Chao P Zhang L Xu L Cui X Wang S. Single-cell RNA and transcriptome sequencing profiles identify immune-associated key genes in the development of diabetic kidney disease. Front Immunol. (2023) 14:1030198. doi: 10.3389/fimmu.2023.1030198 37063851 PMC10091903

[310] Liu S Zhao Y Lu S Zhang T Lindenmeyer MT Nair V. Single-cell transcriptomics reveals a mechanosensitive injury signaling pathway in early diabetic nephropathy. Genome Med. (2023) 15:2. doi: 10.1186/s13073-022-01145-4 36627643 PMC9830686

[311] Wang Y Liu B Zhao G Lee Y Buzdin A Mu X. Spatial transcriptomics: Technologies, applications and experimental considerations. Genomics. (2023) 115:110671. doi: 10.1016/j.ygeno.2023.110671 37353093 PMC10571167

[312] Ferkowicz MJ Verma A Barwinska D Melo Ferreira R Henderson JM Kirkpatrick M. Molecular signatures of glomerular neovascularization in a patient with diabetic kidney disease. Clin J Am Soc Nephrol. (2023) 19:266–75. doi: 10.2215/cjn.0000000000000276 PMC10861111 37533147

